# CaM Kinase II mediates maladaptive post-infarct remodeling and pro-inflammatory chemoattractant signaling but not acute myocardial ischemia/reperfusion injury

**DOI:** 10.15252/emmm.201403848

**Published:** 2014-09-05

**Authors:** Martin Weinreuter, Michael M Kreusser, Jan Beckendorf, Friederike C Schreiter, Florian Leuschner, Lorenz H Lehmann, Kai P Hofmann, Julia S Rostosky, Nathalie Diemert, Chang Xu, Hans Christian Volz, Andreas Jungmann, Alexander Nickel, Carsten Sticht, Norbert Gretz, Christoph Maack, Michael D Schneider, Hermann-Josef Gröne, Oliver J Müller, Hugo A Katus, Johannes Backs

**Affiliations:** 1 Research Unit Cardiac Epigenetics, Department of Cardiology, University of HeidelbergHeidelberg, Germany; 2 DZHK (German Centre for Cardiovascular Research), Partner Site Heidelberg/MannheimHeidelberg, Germany; 3 Department of Cardiology, University of HeidelbergHeidelberg, Germany; 4 Department of Cardiology, Saarland UniversityHomburg, Germany; 5 Medical Research Center, University of Heidelberg Medical Faculty MannheimMannheim, Germany; 6 British Heart Foundation Centre of Research Excellence, Faculty of Medicine, National Heart and Lung Institute, Imperial College LondonLondon, UK; 7 Department of Cellular and Molecular Pathology, German Cancer Research CenterHeidelberg, Germany

**Keywords:** apoptosis, Ca^2+^/calmodulin-dependent protein kinase II, cardiac remodeling, gene replacement, ischemia/reperfusion injury

## Abstract

CaMKII was suggested to mediate ischemic myocardial injury and adverse cardiac remodeling. Here, we investigated the roles of different CaMKII isoforms and splice variants in ischemia/reperfusion (I/R) injury by the use of new genetic CaMKII mouse models. Although CaMKIIδC was upregulated 1 day after I/R injury, cardiac damage 1 day after I/R was neither affected in CaMKIIδ-deficient mice, CaMKIIδ-deficient mice in which the splice variants CaMKIIδB and C were re-expressed, nor in cardiomyocyte-specific CaMKIIδ/γ double knockout mice (DKO). In contrast, 5 weeks after I/R, DKO mice were protected against extensive scar formation and cardiac dysfunction, which was associated with reduced leukocyte infiltration and attenuated expression of members of the chemokine (C-C motif) ligand family, in particular CCL3 (macrophage inflammatory protein-1α, MIP-1α). Intriguingly, CaMKII was sufficient and required to induce CCL3 expression in isolated cardiomyocytes, indicating a cardiomyocyte autonomous effect. We propose that CaMKII-dependent chemoattractant signaling explains the effects on post-I/R remodeling. Taken together, we demonstrate that CaMKII is not critically involved in acute I/R-induced damage but in the process of post-infarct remodeling and inflammatory processes.

## Introduction

CaM Kinase II (CaMKII) is a serine/threonine protein kinase with a broad spectrum of substrates (Braun & Schulman, 1995; Means, 2000). It is activated by Ca^2+^/calmodulin, direct oxidation and a β-arrestin-dependent mechanism (De Koninck & Schulman, 1998; Erickson*et al*, 2008; Mangmool*et al*, 2010; He*et al*, 2011). The four isoforms of CaMKII (α, β, γ, and δ) are encoded by different genes, which display distinct but overlapping expression patterns (Tombes*et al*, 2003). CaMKIIδ and γ are the predominant CaMKII isoforms in the heart with CaMKIIδ displaying the highest level of expression. Alternative splicing mechanisms provide subcellular targeting to locations such as the nucleus (CaMKIIδB) or cytosolic compartments (CaMKIIδC) (Tombes*et al*, 2003). Cardiomyocyte apoptosis was suggested to be mediated through the cytosolic CaMKIIδC splice variant via a mitochondrial cell death pathway (Zhu*et al*, 2003, 2007). Transgenic cardiac overexpression of CaMKIIδC leads to dilated cardiomyopathy (Zhang*et al*, 2003) and arrhythmias (Sossalla*et al*, 2011). The nuclear counterpart CaMKIIδB was shown to be downregulated after myocardial ischemia/reperfusion (I/R) in rats (Peng*et al*, 2010). Interestingly, CaMKIIδB has been even implicated to suppress apoptosis by inducing the anti-apoptotic proteins Bcl-2 and HSP70 (Little*et al*, 2009) and to induce pathological cardiac remodeling by phosphorylation of histone deacetylase 4 (HDAC4) (Backs*et al*, 2006; Zhang*et al*, 2007). In macrophages, CaMKIIγ links ER stress with Fas and mitochondrial apoptosis pathways (Timmins*et al*, 2009), but the specific role of CaMKIIγ in cardiomyocytes has not been investigated. Recently, it was shown that mitochondrial CaMKII can bind and regulate the mitochondrial Ca^2+^ uniporter (MCU), upregulating its activity by phosphorylation of the channel N terminus. Furthermore, transgenic expression of a mitochondrial-targeted CaMKII inhibitory peptide protected against cardiac I/R injury (Joiner*et al*, 2012). In the context of subcellular localization of CaMKII and CaMKIIδ splice variants, it was shown that—despite differences in the quantities of CaMKIIδ splice variants in the nucleus, SR, cytosol, or mitochondria—both δB and δC can be found at some proportion in each compartment (Mishra*et al*, 2011). However, it remains unclear which CaMKII isoform plays the leading role in mitochondria and whether CaMKII levels (or proportions of its splice variants) are regulated during pathological stress such as I/R injury (Correll & Molkentin, 2013).

Global genetic deletion of CaMKIIδ has been shown to attenuate pathological cardiac remodeling induced by transverse aortic constriction (TAC), a model of pressure overload-induced heart failure (Backs*et al*, 2009; Ling*et al*, 2009). However, little is known about the effects of a global CaMKIIδ knockout (KO) in the context of MI or I/R. For example, the biological stress and neurohumoral milieu are markedly different in acute ischemic injury and later adaptation to infarction, and both differ from a simple increase in after load. Recently, it was demonstrated that CaMKIIδ mediates nuclear factor-κB (NF-κB) activation in cardiomyocytes after*in vivo* I/R in mice and suggested that CaMKIIδ serves to trigger changes in inflammatory gene expression that contribute to myocardial I/R damage. In this study, CaMKIIδ KO mice were protected against I/R damage as evidenced by decreased infarct size, attenuated apoptosis, and improved functional recovery (Ling*et al*, 2013). However, it remains unclear to what extent redundant effects mediated by CaMKIIγ play a role in the heart, as both isoforms, δ and γ, are activated in response to chronic stress (Colomer*et al*, 2003). In addition, the specific roles of CaMKIIδ splice variants δB and δC in the context of I/R injury have not been investigated yet. Other studies addressing the role of CaMKII in myocardial infarction (MI) or I/R have been reported. Protective effects of CaMKII inhibition have also been observed in experiments using non-genetic approaches, for example, overexpression of peptides or pharmacologic compounds such as KN-93 (Zhang*et al*, 2005; Yang*et al*, 2006; Vila-Petroff*et al*, 2007; Joiner*et al*, 2012). These approaches face two limitations: (i) The inhibition of CaMKII, such as assessed by phospholamban phosphorylation, was not complete (Zhang*et al*, 2005). (ii) Off-target effects, for example, on protein kinase D and ion channels have been observed (Gao*et al*, 2006; Rezazadeh*et al*, 2006; Backs*et al*, 2009). Therefore, we aimed to investigate the essential and specific roles of the cardiac CaMKII isoforms using new genetic models. We used a clinically relevant*in vivo* paradigm of myocardial I/R, closely resembling the patient setting of acute MI and revascularization therapy.

## Results

### CaMKIIδ does not regulate myocardial damage shortly after I/R injury

**Figure 1:**
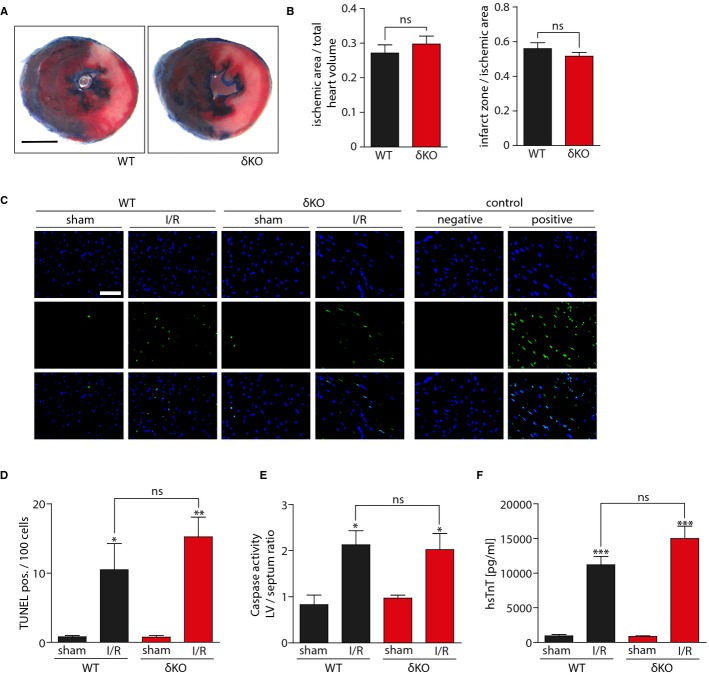
CaMKIIδ deletion does not affect infarct size in ischemia/reperfusion injury I/R surgery was performed in Camk2δ^−/−^ C57BL/6 (δKO) and wild-type C57BL/6 (WT) mice. Transverse heart sections were obtained from WT and δKO mice 24 h after myocardial I/R injury. All sections were stained with Evans Blue and TTC. Representative images are shown. Scale bar: 2 mm.Quantitative measurement of ischemic area/total heart volume ratio and infarct zone/ischemic area ratio was performed. Planimetrically and volumetrically calculated infarct sizes show no significant differences.*N* = 8 animals operated and analyzed in δKO group, 10 animals in WT group. Infarct zone/ischemic area: ns,*P* = 0.33. Ischemic area/total heart volume: ns,*P* = 0.45.Representative photomicrographs showing TUNEL staining of WT and δKO left ventricular (LV) transverse sections 24 h after myocardial I/R injury or sham operation. Positive: pretreatment with DNase I; negative: without TUNEL enzyme solution. Scale bar: 50 μm. (upper) DAPI, (middle) TUNEL, (lower) merge.Quantification of TUNEL-positive cells per 100 DAPI-positive nuclei.*N* = 5 animals per group. ns,*P* = 0.37; *P = 0.04; **P = 0.01.Caspase-3/7 enzyme activity was measured in LV homogenates. Values are normalized to caspase-3/7 activity in septal myocardium.*N* = 3 animals per group. ns,*P* = 0.84; *P = 0.02 in WT group; *P = 0.04 in δKO group.High-sensitive serum Troponin T (hsTnT) was measured 24 h after myocardial I/R injury.*N* = 5 animals in WT sham group, 11 animals in WT I/R group, 10 animals in δKO sham group, 8 animals in δKO I/R group. ns,*P* = 0.08; ***P = 0.0007 in WT group; ***P = 0.0001 in δKO group. Data information: All data are expressed as mean ± SEM. Unpaired Student's*t*-test (B) and one-way ANOVA with Bonferroni's multiple comparison test (D–F) were used to compare groups. ns = non-significant.

To determine whether the most abundant cardiac CaMKII isoform, CaMKIIδ, is critical for myocardial I/R injury, we used CaMKIIδ-deficient mice (δKO). We used the same time course for I/R injury in our KO mouse model as it was used before (LAD occlusion for 60 min followed by 24 h reperfusion) (Ling*et al*, 2013). The ischemic area, normalized to total heart volume, did not significantly differ between wild-type (WT) and δKO mice, as assessed by Evans Blue staining. In contrast to the recent report (Ling*et al*, 2013), no difference was observed in the size of whitened triphenyltetrazolium chloride (TTC)-stained infarct zone/ischemic area percentage (Fig1A and B). We also determined the extent of apoptotic cell death after myocardial I/R injury by TUNEL assays. Hearts from mice that underwent I/R surgery showed a distinct increase of TUNEL-positive cells as compared to sham controls, but no difference between WT and δKO animals could be detected (Fig1C and D). Consistently, I/R led to a similar increase in caspase-3/7 enzyme activity in WT and δKO mice (Fig1E). Because both apoptosis and necrosis mediate cell death in MI and I/R (Saraste*et al*, 1997; James, 1998; McCully*et al*, 2004), we also measured high-sensitive cardiac Troponin T (hsTnT) serum levels as a marker for myocardial cell death. Repeated measurements of serum hsTnT at defined time points after I/R surgery in a group of WT mice showed that hsTnT serum time course was similar to serum kinetics observed in patients after MI (Supplementary Fig S1A) (Katus*et al*, 1991). In addition, a linear correlation between hsTnT serum levels and planimetrically calculated infarct sizes was observed (Supplementary Fig S1B). Based on these results, serum hsTnT measurements were performed in all animals 24 h after I/R surgery. Again, no significant difference was observed between WT and δKO mice (Fig1F), indicating that the global deletion of CaMKIIδ does not affect myocardial damage shortly after I/R. In order to test for infarct size differences at an earlier time point of ischemic injury, we performed I/R experiments to compare I/R damage in WT and CaMKIIδKO mice after only 30 min of ischemia. Again, no significant difference between the groups could be found (Supplementary Fig S2). To confirm, that the chosen experimental setup is able to detect changes in acute I/R injury caused by other interventions, we pretreated one group with enalapril as reported before (Leuschner*et al*, 2010). As expected, enalapril led to a significant reduction of infarct size (Supplementary Fig S3).

### Role of CaMKIIδ splice variants in acute myocardial I/R injury

**Figure 2:**
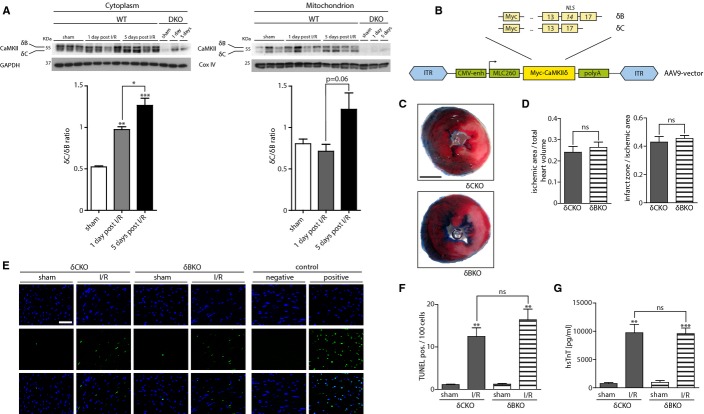
CaMKIIδ splice variants δB and δC are not critical for acute I/R injury CaMKII expression after I/R injury. WT mice underwent surgery and were challenged by 60 min of ischemia followed by either 24 h or 5 days of reperfusion. LV homogenates were further processed to obtain cytoplasmic and mitochondrial fractions. (Top) CaMKII expression levels as detected by Western blot analysis are shown. Upper band represents CaMKIIδB, lower band CaMKIIδC splice variant (Bottom). Bar graphs quantify splice variant shift, depicted as δC/δB ratio.*N* = 3 in sham groups, 4 in I/R groups. *P = 0.04; **P = 0.01 versus sham; ***P = 0.0009 versus sham. To confirm the specificity of the CaMKII band, LV homogenates of mice lacking the two CaMKII genes delta and gamma (DKO, see also Fig 3) were loaded.AAV9 vectors containing myc-tagged CaMKIIδ splice variants δB or δC were designed and produced. Numbers in boxes represent CaMKIIδ exon numbers. NLS = nuclear localization sequence (exon 14), ITR = inverted terminal repeat sequence, CMV-enh = cytomegalovirus enhancer, MLC260 = myosin light chain 260 promoter, polyA = polyadenylation tail. Camk2δ^−/−^ mice received a single tail vein injection of 5 × 10^11^vg AAV9-CaMKIIδB (δB) or AAV9-CaMKIIδC (δC). I/R surgery was performed after 4 weeks.Transverse heart sections were obtained from mice expressing CaMKIIδB or δC at 24 h after myocardial I/R injury. All sections underwent Evans Blue and TTC staining. Representative images are shown. Scale bar: 2 mm.Quantitative measurements of ischemic area/total heart volume ratio and infarct zone/ischemic area ratio were performed. Planimetrically and volumetrically calculated infarct sizes did not differ significantly: ns,*P* = 0.91. Ischemic area/total heart volume: ns,*P* = 0.54.*N* = 8 animals per group operated and analyzed in δCKO group, 7 animals in δBKO group.Representative photomicrographs showing TUNEL staining in δCKO and δBKO LV transverse sections 24 h after myocardial I/R injury or sham operation. Positive: pretreatment with DNase I; negative: without TUNEL enzyme solution. Scale bar: 50 μm. (upper) DAPI, (middle) TUNEL, (lower) merge.Bar graphs show quantification of TUNEL-positive cells per 100 DAPI-positive nuclei.*N* = 3 animals in sham groups, 4 animals in I/R groups. ns,*P* = 0.27; **P = 0.006 in δCKO group; **P = 0.004 in δBKO group.hsTnT was measured 24 h after myocardial I/R injury.*N* = 4 animals in sham groups, 8 animals in δCKO group, 7 animals δBKO group. ns,*P* = 0.94; **P = 0.002; ***P = 0.0006. Data information: All data are expressed as mean ± SEM. Unpaired Student's*t*-test (D) and one-way ANOVA with Bonferroni's multiple comparison test (A, F and G) were used to compare groups. ns = non-significant. Source data are available online for this figure.

**Figure 3:**
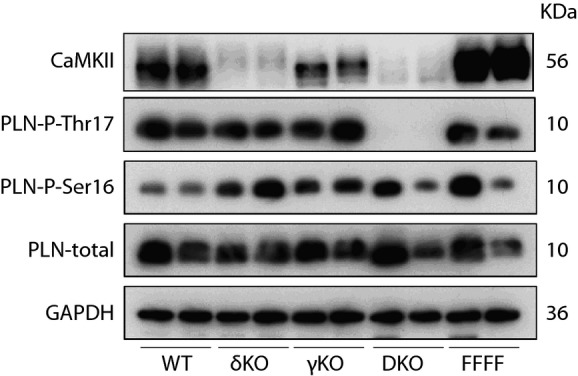
CaMKIIδ and γ KO mouse models To rule out redundant effects of myocardial CaMKIIγ isoform, cardiomyocyte-specific CaMKIIγ/δ double KO mice were created [Camk2γ^loxP/loxP^; Camk2δ^loxP/loxP^; α-MHC-Cre (DKO) and Camk2γ^loxP/loxP^; Camk2δ^loxP/loxP^ (FFFF)]. LV homogenates were subjected to Western blot analysis using antibodies directed against CaMKII, phospho-PLN-Thr17, phospho-PLN-Ser16, PLN, and GAPDH. Although CaMKII expression at the protein level is decreased in both CaMKII delta single KO animals (δKO) and DKO, significant reduction of CaMKII-phosphorylated phospholamban (PLN) at Thr17 can only be seen in DKO hearts. Total amounts of PLN, GAPDH, and PLN at the PKA phosphorylation site Ser16 served as loading control. γKO: CaMKII gamma single KO; WT: wild-type.Source data is available online for this figure.

Recently, it has been shown that in a rat animal model, shortly after I/R injury, expression of CaMKIIδ splice variants δB and δC are inversely regulated, with CaMKIIδC showing increased expression levels (Peng*et al*, 2010). In order to investigate expression of CaMKII and its splice variants after mouse I/R injury, a group of WT animals underwent*in vivo* I/R surgery. After reperfusion periods of either 1 or 5 days, hearts were harvested to analyze CaMKII expression by Western blot. Because it was shown that mitochondrial-targeted CaMKII inhibition protects against myocardial I/R injury (Joiner*et al*, 2012), we analyzed CaMKII expression levels both in mitochondria and the cytoplasm. As depicted in Fig2A, 24 h after I/R surgery, a shift in cytoplasmic CaMKIIδ splice variant expression toward increased levels of CaMKIIδC could be detected, which was emphasized after 5 days. Interestingly, we could not detect any shift or increased expression levels of total CaMKII in the mitochondrial fraction after 1 day. However, there was a trend toward increased CaMKIIδC expression levels after 5 days. The purity of the mitochondrial fraction was confirmed by Western blot analysis against Cox IV (Supplementary Fig S4). In the available CaMKIIδ KO mice (Backs*et al*, 2009; Ling*et al*, 2009, 2013), CaMKIIδ is deleted along with its splice variants including δB and δC. Because it was suggested that δB and δC play opposite roles in the context of ischemic stress (Maier*et al*, 2003; Zhu*et al*, 2003, 2007; Little*et al*, 2009; Peng*et al*, 2010), and considering the shift in splice variant expression after I/R injury as described above, we hypothesized that the specific deletion of the δC splice variant might protect the heart against acute I/R injury. Thus, we took advantage of the cardiotrophic adeno-associated virus 9 (AAV9)*in vivo* expression system (Goehringer*et al*, 2009) and re-expressed δB and δC under the control of a CMV-enhanced MLC260 promotor (Geisler*et al*, 2011; Schinkel*et al*, 2012) in the CaMKIIδ-KO background, which resulted in a functional KO of the non-expressed counterpart (Fig2B). This enabled us to titrate the AAV9-MLC260-δC and AAV9-MLC260-δB virus concentrations to a degree that allowed expression close to endogenous CaMKII levels (Supplementary Fig S5A). Doing so, we could reduce the likelihood that unspecific effects occur as discussed for the alternative overexpression approach by the use of transgenic CaMKIIδB/CaMKIIδC mice (Zhang*et al*, 2002, 2003). Based on the titration experiment, we next injected 5 × 10^11^ virus particles of AAV9-MLC260-CaMKIIδB (δCKO) or AAV9-MLC260-CaMKIIδC (δBKO) into CaMKIIδ-KO mice to create mice specifically lacking only one of the two splice variants. At baseline, AAV9-injected mice did not differ with respect to cardiac function, assessed by echocardiography (Supplementary Fig S5B). Then, these mice underwent I/R surgery. As depicted in Supplementary Fig S5C, CaMKIIδC splice variant was significantly upregulated after 60 min of ischemia and 24 h of reperfusion, while there was only a trend toward an upregulation of CaMKIIδB splice variant. In order to test CaMKII function of AAV9 splice variants, we expressed CaMKIIδB and CaMKIIδC splice variants in CaMKIIδ KO mice and measured PLN-P-Thr17 phosphorylation as indicator for CaMKII activity by Western blot analyses. Reduced PLN phosphorylation at Thr17 in CaMKIIδ KO samples was restored by CaMKIIδ splice variant expression (Supplementary Fig S5D). Surprisingly, quantification of ischemic areas, normalized to total heart volumes, revealed no significant differences in areas at risk between δCKO and δBKO mice after I/R injury. Moreover, infarct sizes did not differ between the groups (Fig2C). Consistently, no significant differences were observed in apoptotic or necrotic markers, analyzed by TUNEL-staining and serum hsTnT measurements (Figs2D and 3F).

### Combined cardiomyocyte-specific deletion of CaMKIIδ/γ does not affect I/R injury

To test the possibility that CaMKIIγ compensates for CaMKIIδ, we aimed to use CaMKIIδ/γ double KO mice. Because the combined global deletion of CaMKIIδ and γ resulted in early postnatal lethality, we generated double-floxed CaMKIIδ/γ mice (CaMKIIγ^loxP/loxP^; CaMKIIδ^loxP/loxP^, here termed as FFFF) and crossed them with mice expressing Cre-recombinase under the control of the cardiomyocyte-specific α-MHC-promoter (Agah*et al*, 1997) to receive cardiomyocyte-specific CaMKIIδ/γ double KO mice (DKO). To determine the degree of CaMKII deficiency, we performed Western blot analysis with ventricular protein extracts using an antibody directed against CaMKIIδ and γ (Backs*et al*, 2009, 2010), and against the well-characterized CaMKII-phosphorylation site Thr-17 of phospholamban (PLN) to estimate CaMKII activity (Fig3). The deletion of CaMKIIδ led to a dramatic reduction of CaMKII protein but only to a moderate loss of PLN-Thr17 phosphorylation, indicating that CaMKIIγ, which we found to contribute only to a very small degree to the detectable CaMKII level, compensates for CaMKIIδ. Consistent with this hypothesis, DKO hearts showed a dramatic loss in PLN-Thr17 phosphorylation. The residual CaMKII signal in DKO likely reflects CaMKII expression in non-cardiomyocytes. Thus, we also confirmed these differences between single and double KO mouse models in isolated adult cardiomyocytes (Supplementary Fig S6).

**Figure 4:**
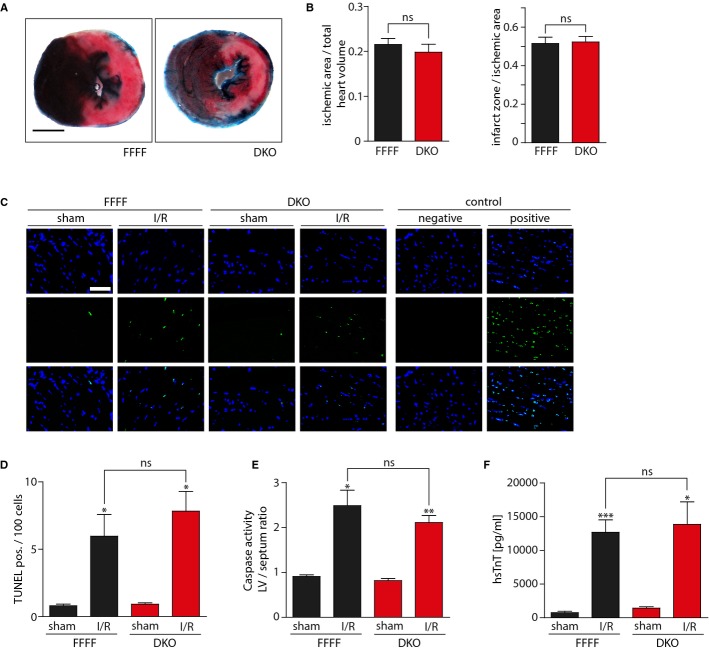
Combined cardiomyocyte-specific deletion of CaMKIIδ/γ does not affect I/R injury DKO mice underwent I/R surgery [Camk2γ^loxP/loxP^; Camk2δ^loxP/loxP^; α-MHC-Cre (DKO) versus Camk2γ^loxP/loxP^; Camk2δ^loxP/loxP^ (FFFF)]. Images of representative transverse heart sections after Evans Blue and TTC staining. Scale bar: 2 mm.Planimetrically and volumetrically calculated infarct sizes show no significant differences.*N* = 9–13 animals operated and analyzed per group. ns,*P* = 0.86. Ischemic area/total heart volume: ns,*P* = 0.48.Representative photomicrographs of TUNEL-stained LV transverse sections after I/R or sham surgery. Positive: pretreatment with DNase I; negative: without TUNEL enzyme solution. Scale bar: 50 μm. (upper) DAPI, (middle) TUNEL, (lower) merge.Bar graphs show quantification of TUNEL staining.*N* = 3 animals per sham group, 4 animals in FFFF group, 5 animals in DKO group. ns,*P* = 0.42; *P = 0.04 in FFFF group; *P = 0.01 in DKO group.Caspase-3/7 enzyme activity was measured in LV homogenates.*N* = 2 animals per sham group, 3 animals in FFFF group, 4 animals in DKO group. ns,*P* = 0.32; *P = 0.04; **P = 0.006.hsTnT was measured 24 h after ischemia.*N* = 5 animals per sham group, 9 animals in FFFF group, 13 animals in DKO group. ns,*P* = 0.79; *P = 0.04; ***P = 0.0005. Data information: Data are expressed as mean ± SEM. Unpaired Student's*t*-test (B) and one-way ANOVA with Bonferroni's multiple comparison test (D–F) were used to compare groups. ns = non-significant.

We then performed I/R surgery in FFFF and DKO mice. Despite the complete loss of CaMKII activity in DKO, infarct size quantification of Evans Blue- and TTC-stained transverse sections revealed no significant differences between FFFF and DKO (Fig4A and B). Consistent with the macroscopic findings, no significant differences were observed in apoptotic or necrotic markers, as analyzed by TUNEL-staining, caspase-3/7 activity measurements and serum hsTnT measurements (Fig4C–F).

### Inducible CaMKIIδ and γ deletion does not protect against acute I/R injury

Given the high number of related cardiac signaling pathways involved in cellular responses to MI (Pfeffer & Braunwald, 1990; Kehat & Molkentin, 2010), the possibility exists that another pathway compensates for CaMKIIδ/γ in the context of ischemia, which might not be reflected by PLN-Thr17 phosphorylation. Because compensation occurs more likely in congenital loss-of-function models, we sought to investigate the effects of I/R in an inducible DKO mouse model. Thus, we directed expression of Cre-recombinase to the adult heart using cardiotrophic AAV9 vectors. In this vector (AAV9-MLC260-Cre), Cre is expressed under the control of a CMV-enhanced MLC260 promotor (Supplementary Fig S7A). By this method, we achieved after 12 weeks a substantial CaMKII downregulation and a clear reduction in PLN-Thr17 phosphorylation by a single intravenous (i.v.) injection of AAV9-MLC260-Cre into FFFF (Supplementary Fig S7B and C). In unstressed FFFF mice, the AAV9 treatment did not affect cardiac function assessed by echocardiography (Supplementary Fig S7D). We then performed I/R surgery on FFFF mice 12 weeks after treatment with AAV9-MLC260-Cre (iDKO) and as a control 12 weeks after treatment with AAV-MLC260-Luc (expression of luciferase under the control of MLC260). By Western blot analysis, we confirmed a sufficient CaMKII take out in all experimental iDKO mice. But again, assessment of infarct size did not demonstrate any significant difference of infarct zone/ischemic area ratios between the groups (Supplementary Fig S7E and F). Moreover, no significant differences were observed in apoptotic or necrotic markers, as analyzed by TUNEL-staining and serum hsTnT measurements (Supplementary Fig S7G–I).

### CaMKII DKO mice show a reduced infarct size and improved cardiac function 5 weeks after I/R surgery

**Figure 5:**
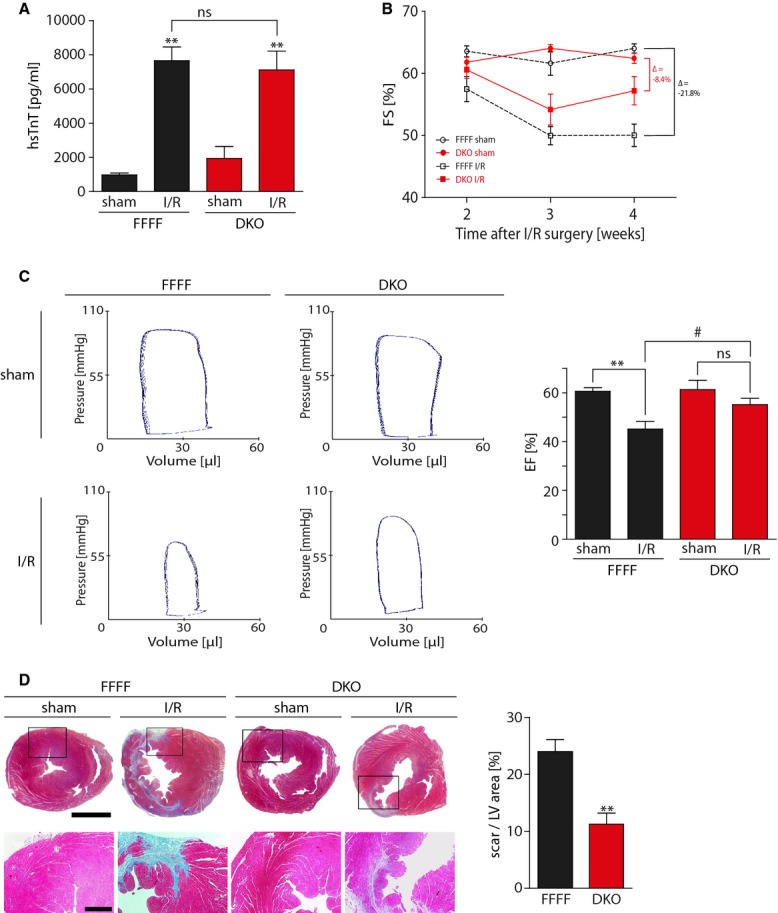
Improved cardiac function and reduced scar formation in DKO 5 weeks after I/R surgery DKO and FFFF littermates were subjected to I/R with 5 weeks of follow-up. Echocardiography was performed at 2, 3 and 4 weeks post-surgery. At 5 weeks, invasive hemodynamic parameters were assessed and hearts were harvested for histological analysis. hsTnT was measured 24 h after myocardial I/R injury and revealed equal infarcts sizes in DKO and FFFF at this time point.*N* = 6 animals in sham groups, nine animals in FFFF I/R group, 10 animals in DKO I/R group. ns,*P* = 0.68; **P = 0.001 in FFFF group; **P = 0.003 in DKO group.Transthoracic echocardiography revealed a significant difference in fractional shortening (FS) between FFFF and DKO 4 weeks after I/R surgery.*N* = 12 animals in FFFF sham group, 11 animals in DKO sham groups, 13 animals in I/R groups.Representative pressure–volume loops showing improved hemodynamic cardiac function in DKO as compared to FFFF controls. Cardiac ejection fraction (EF) was assessed and revealed a decrease in EF from 60.6 ± 4.4 to 44.1 ± 10.5 in FFFF mice after I/R surgery. No significant decrease in EF in DKO mice (61.3 ± 11.4 to 53.5 ± 10.6) was observed.*N* = 9 animals per sham group, 13 animals per I/R group. ns,*P* = 0.11; **P = 0.004; ^#^*P* = 0.04.Representative images showing Masson's trichrome staining of transverse cardiac sections. Rectangular selections are shown in greater magnification below. Section plane: 3 mm above the heart apex. Scale bars: 2 mm (upper) and 500 μm (lower). Bar graphs show infarct size quantification expressed as scar area per LV area.*N* = 5 animals in FFFF group, 7 animals in DKO group. **P = 0.001. Data information: Data are expressed as mean ± SEM. Unpaired Student's*t*-test (D) and one-way ANOVA with Bonferroni's multiple comparison test (A and C) were used to compare groups. ns = non-significant.

We next tested the hypothesis that genetic deletion of cardiac CaMKII protects the heart only in a long-term follow-up I/R study. Therefore, we performed I/R surgery on DKO and FFFF mice according to an extended protocol of 60 min LAD occlusion, followed by 5 weeks of reperfusion. To rule out surgery related differences of infarct sizes between the groups, we obtained blood for serum hsTnT measurements 24 h after surgery, confirming similar infarct sizes in the experimental groups shortly after I/R injury (Fig5A). We then performed echocardiography 2, 3 and 4 weeks after I/R surgery to assess cardiac function. Strikingly, FFFF I/R mice showed a progressive deterioration of cardiac function from 2 to 4 weeks post-surgery, while this functional impairment was ameliorated in DKO mice after 4 weeks (Fig5B). At 5 weeks after I/R injury, all mice were subjected to hemodynamic characterization. Invasive parameters clearly indicate a significantly higher ejection fraction (EF) in DKO mice compared to FFFF controls (Fig5C and Table1). Histological infarct size quantification revealed reduced scar formation in response to I/R in DKO as compared to FFFF controls. Moreover, the degree of fibrosis, as seen at higher magnification, was apparently less dense in DKO (Fig5D). Taken together, these findings most likely account for improved cardiac function in DKO mice. Similar data were obtained in an independent experiment with a follow-up of 6 weeks after surgery. We followed these mice by echocardiography with a Visual Sonics Vevo 2100 instrument to measure endocardial fractional area change (FAC), which represents cardiac function and specifically detects regional contraction deficits due to infarct scars. Here, we found again an improved cardiac function of DKO mice at 6 weeks after I/R surgery, as compared to FFFF controls. In addition, we repeated invasive pressure-volume analyses, confirming increased ejection fraction and decreased dilation parameters in DKO hearts (Supplementary Fig S8).

**Table 1:** Morphologic features and hemodynamic analysis of CaMKII DKO and FFFF mice, 5 weeks after I/R surgery

	FFFF		DKO	
	Sham	I/R	Sham	I/R
Heart weight (mg)	125.0 ± 16.0	127.0 ± 12.5	144.0 ± 22.4	139.7 ± 13.8
Body weight (g)	27.2 ± 2.1	26.7 ± 2.1	27.5 ± 2.9	26.0 ± 2.9
HW/BW ratio (mg/g)	4.6 ± 0.3	4.4 ± 1.4	5.2 ± 0.4	5.2 ± 0.4
Heart rate (bpm)	358.4 ± 29.5	360.0 ± 34.5	401.4 ± 30.2	390.3 ± 40.1
Ejection fraction (%)	60.6 ± 4.4	44.1 ± 10.9†	61.3 ± 11.4	53.5 ± 10.6‡
*dP*/*dt*_max_ (mmHg/s)	5,514 ± 1300	4,477 ± 1993	6,065 ± 989	5,702 ± 1661
*dV/dt*_max_ (μL/s)	1,360 ± 546	1,295 ± 486	1,536 ± 498	1,445 ± 531
LVESP (mmHg)	103.3 ± 13.2	83.6 ± 12.0†	105.0 ± 14.8	93.6 ± 9.2‡
LVEDP (mmHg)	3.6 ± 1.3	3.6 ± 1.9	2.8 ± 0.9	3.3 ± 1.2
SV (μL)	25.1 ± 4.4	20.1 ± 5.8*	25.8 ± 5.5	24.7 ± 9.2
Tau (ms)	11.6 ± 2.2	12.1 ± 2.0	9.8 ± 1.1	11.6 ± 3.0

HW/BW, heart weight/body weight ratio; LVESP, LV end-systolic pressure; LVEDP, LV end-diastolic pressure; SV, stroke volume; Tau, time constant of pressure relaxation.

CaMKII DKO and FFFF mice were subjected to hemodynamic detection 5 weeks after I/R surgery. Data are expressed as mean ± SD of values from nine mice in sham groups and 13 mice in I/R groups.

* *P *< 0.05.

† *P *< 0.01 versus sham FFFF.

‡ *P *< 0.05 versus I/R FFFF.

### CaMKII mediates cardiac leukocyte infiltration and chemoattractant signaling

**Figure 6:**
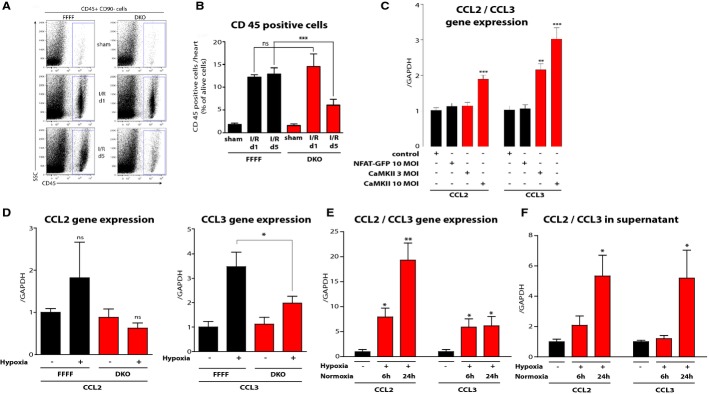
CaMKII mediates infiltration o CD45^+^ cells and pro-inflammatory chemoattractant signaling Representative dot plots of hearts analyzed by flow cytometry comparing sham operated, I/R d1 and I/R d5 from DKO and FFFF control animals. Blue gates indicate CD45^+^ cells.*N* = 5 animals per sham group, 6 animals per I/R group.Quantitative analysis. Reduced infiltration of CD45^+^ leukocytes occurs 5 days after I/R injury in DKO mice, while no significant differences can be detected on day 1 after I/R injury. ns,*P* = 0.86; ***P = 0.0004.Gene expression as determined by real-time RT–PCR of CCL2 and CCL3 in NRVMs under control conditions and after adenoviral infection with active CaMKII (contains T287D mutation for auto-activation) or NFAT-GFP as additional control. Gene expression is normalized to GAPDH.*N* = 6 independent experiments per group. ***P = 0.0001 in CCL2 group; **P = 0.002; ***P =* *0.0001 in CCL3 group.Gene expression as determined by real-time RT–PCR of CCL2 and CCL3 in adult mouse ventricular myocytes (AMVMs) from FFFF and DKO mice (*n* = 6 wells per condition) after hypoxia, normalized to GAPDH. ns,*P* = 0.68 in FFFF group; ns,*P* = 0.29 in DKO group; *P = 0.01.Gene expression as determined by real-time RT–PCR of CCL2 and CCL3 in NRVMs after hypoxia followed by normoxia periods as indicated, normalized to GAPDH, respectively.*N* = 4 wells per condition. *P = 0.02; **P = 0.006 in CCL2 group; *P = 0.01 (6 h); *P = 0.02 (24 h) in CCL3 group.Quantification of CCL2 and CCL3 protein as determined by ELISA in supernatant from NRVMs after hypoxia followed by normoxia periods as indicated.*N* = 3 per condition. *P = 0.03 in CCL2 group; *P = 0.04 in CCL3 group. Data information: Data are expressed as mean ± SEM. One-way ANOVA with Bonferroni's multiple comparison test was used to compare groups. ns = non-significant.

Next, we hypothesized that the attenuation of post-infarct remodeling might be mediated by inflammatory processes. In order to investigate the kinetics of leukocyte infiltration after I/R injury, we performed flow cytometric analyses in a time course of one and 5 days after I/R surgery. We observed reduced infiltration of CD45^+^ leukocytes 5 days after I/R injury in DKO mice, while no significant differences were observed 1 day after I/R injury, possibly explaining the late effects of CaMKII deletion (Fig6A and B). To identify CaMKII-dependent inflammatory pathways, we performed unbiased and systematic RNA analyses by Affymetrix chip arrays in cardiac samples at 1 and 5 days after I/R injury versus sham-operated hearts. Gene Set Enrichment Analysis (GSEA) was then used to determine whether defined sets of genes exhibit a statistically significant bias in their distribution within a ranked gene list (Subramanian*et al*, 2005). Pathways belonging to specific cell functions were obtained from public external databases (KEGG PATHWAY Database, http://www.genome.jp/kegg/) to perform pathway analyses. Doing so, we identified several inflammatory pathways to be attenuated in DKO as compared to FFFF (Supplementary Fig S9A). Intriguingly, the “Chemokine Signaling Pathway” was attenuated at both 1 and 5 days after I/R. Whereas several ligands to inflammatory cells were attenuated 1 and 5 day after I/R, the expression of intracellular components of inflammatory cells were mostly attenuated 5 days after I/R, confirming an attenuated infiltration of inflammatory cells at this time point (Supplementary Fig S9B). We then validated the top 5 attenuated genes of the “Chemokine Signaling Pathway” at the 1 and 5 day time point after I/R by RT–PCR (Supplementary Fig S9C), and we could confirm an attenuated gene expression of CCL3, CCR1, CXCR2, PPBP, HCK, FGR, and CCL6. However, CXCL3 could not be detected by RT–PCR and VAV1 and CCL2 could not formally be validated, although a not statistically significant trend toward attenuated expression could be noted. Based on the observation that 5 days after I/R more intracellular components of inflammatory cells were enriched, we speculated that these changes are rather indirectly and secondarily mediated by CaMKII by non-cardiomyocyte autonomous effects. Vice versa, we envisioned that the chemoattractant ligands, which were already attenuated 1 day after I/R, might be induced by CaMKII in cardiomyocytes. Thus, we tested whether adenoviral expression of active CaMKII (CaMKII-T287D) was sufficient to induce mRNA expression of selected genes of the “Chemokine Signaling Pathway”. Strikingly, CaMKII induced only expression of members of the chemokine (C-C motif) ligand family (Fig6C), in particular CCL2 (also known as monocyte chemotactic protein 1, MCP1) and even more pronounced CCL3 (also known as macrophage inflammatory protein 1 alpha, MIP-1 alpha) but not CCR1, VAV1, HCK, or FGR (Supplementary Fig S10), which according to the KEGG PATHWAY Database are all intracellular components of inflammatory cells, supporting our hypothesis that these genes are rather indirectly and secondarily regulated by CaMKII via non-cardiomyocyte autonomous effects. To rule out an artificial effect due to adenovial infections of NRVMs, we used another adenovirus with a high titer (10 MOI), in this case NFAT-GFP, as a control and found no effect on CCL2 and CCL3 expression. In these experiments, CaMKII expression was confirmed by RT–PCR (3 MOI led to a 18-fold increase in CaMKII expression and 10 MOI to a 114-fold increase).

To further support these data, we asked whether CaMKII is also required for CCL2 and CCL3 gene expression in isolated cardiomyocytes. Thus, we isolated adult mouse ventricular myocytes (AMVMs) from FFFF and DKO and applied hypoxia followed by 8 h normoxia (Fig6D). We found that CCL3 but not CCL2 was significantly upregulated by hypoxia, which was clearly attenuated in DKO cardiomyocytes. Taken together, CaMKII is sufficient and required for cardiomyocyte autonomous CCL3 expression. To test whether an increased gene expression of CCL2 and CCL3 results in an increased secretion of the protein, we performed hypoxia experiments in NRVMs (Fig6E and F) and found that an increase in CCL2 and CCL3 mRNA preceded an actual increase of CCL2 and CCL3 protein in the supernatant of NRVMs 24 h after hypoxia, potentially explaining the late effects on infiltration of CD45^+^ cells.

## Discussion

The present study investigated for the first time the specific roles of CaMKIIδ, its splice variants δB and δC, and potential redundant roles of CaMKIIδ and CaMKIIγ in the acute setting of*in vivo* I/R injury and post-infarct remodeling. The important new finding of this work is that CaMKII mediates maladaptive post-infarct remodeling not through a reduction of acute myocardial damage after I/R injury as reported by others.

### CaMKII loss-of-function models

Because of the lack of sufficient CaMKII loss-of-function models, one could not comprehensively study the essential roles of the CaMKII genes and splice variants in the context of cardiovascular disease. Therefore, we focused first on the development of genetic mouse models, in which the cardiac CaMKII genes and splice variants were specifically targeted or re-expressed close to endogenous levels*in vivo*. These models were then combined with a clinically relevant*in vivo* I/R mouse model, resembling a common clinical course of events in patients with MI who undergo revascularization therapy. In contrast to previously described approaches aiming at inhibiting CaMKII, the models established for this study are specific and result—in case of DKO—in a profound and almost complete inhibition of CaMKII activity. The DKO model is so far the most effective approach to fully inhibit cardiac CaMKII activity*in vivo*. Other published*in vivo* models that use either chemical compounds or peptides or single CaMKII KOs did not result in a comparable CaMKII loss of function as shown here by PLN-Thr17 hypo-phosphorylation (Zhang*et al*, 2005; Yang*et al*, 2006; Backs*et al*, 2009; Ling*et al*, 2009, 2013). We also established an inducible DKO model, which we currently also use to further evaluate CaMKII as a therapeutic target in cardiovascular disease by deleting CaMKII after onset of disease. The AAV9-Cre driven gene take out did neither induce AAV- nor Cre-associated cardiomyopathy as it was reported for an alternative approach, in which tamoxifen is used in combination with Mer-Cre-Mer transgenic mice (Koitabashi*et al*, 2009; Molkentin & Robbins, 2009). However, we cannot formally rule out that in the DKO or iDKO model, CaMKIIβ may compensate for the loss of CaMKIIδ and CaMKIIγ. Moreover, we established a CaMKII gene replacement strategy to study the specific functions of the CaMKIIδ splice variants δB and δC. This is of particular interest in the context of I/R injury because it was suggested that δC plays rather a maladaptive and δB rather a protective role in this process (Zhang*et al*, 2003, 2010; Zhu*et al*, 2003, 2007; Little*et al*, 2009; Peng*et al*, 2010). Thus, we were interested to test whether the specific lack of CaMKIIδC protects the heart against acute myocardial I/R injury. We took advantage of the AAV9-mediated expression approach to re-express the CaMKII splice variants at endogenous rather than at artificial overexpression levels. In this regard, CMKIIδB and CMKIIδC transgenic mice showed at least a 10-fold overexpression of CaMKII in the heart (Zhang*et al*, 2002, 2003), which exceeds the expression levels as observed in pathological conditions (see Fig2).

### CaMKII in acute I/R injury

Previous work suggested that CaMKII inhibition prevents adverse remodeling following MI (Zhang*et al*, 2005). Furthermore, evidence was provided that CaMKII mediates apoptosis in the acute setting of MI (Yang*et al*, 2006; Joiner*et al*, 2012). In these studies, CaMKII inhibition was achieved by cardiac expression of a CaMKII inhibitory peptide, which may also exert CaMKII-independent effects (Backs*et al*, 2009). Therefore, the possibility exists that the inhibition of other signaling molecules accounts for the early anti-apoptotic effects. In another study, pharmacological CaMKII inhibition resulted in smaller infarct sizes and significant reductions of apoptotic and necrotic markers in acute I/R of isolated Langendorff-perfused rat hearts (Vila-Petroff*et al*, 2007). Here, KN-93 was used as pharmacological inhibitor of CaMKII. Although commonly used, KN-93 was also shown to induce general toxicity and to lack specificity for CaMKII (Means, 2000; Hudmon & Schulman, 2002; Gao*et al*, 2006; Rezazadeh*et al*, 2006).

The first study to report protective effects in a genetic CaMKIIδ mouse model in acute I/R injury was published very recently (Ling*et al*, 2013). However, although we used the same protocol of 60 min of LAD ligation followed by 24 h of reperfusion, we could not demonstrate beneficial effects in this setting. A possible explanation for these contradictory results could be found in different genetic backgrounds or different KO strategies. Deletion of CaMKIIδ and CaMKIIγ in the present study was achieved by targeting exons 1 and 2 of the two CaMKII genes, resulting in no residual translation of partial CaMKII peptides (Backs*et al*, 2009). In contrast, the other CaMKIIδ-KO model (Ling*et al*, 2009, 2013) was generated by targeting exons 9 to 11. Thus, the possibility exists that a residual N-terminal CaMKII polypeptide is produced which exerts a dominant-negative effect on CaMKIIγ or modulatory effects on other kinases because the N-terminal part would be predicted to bind to CaMKII phospho-sites (which are often shared with other kinases) but would not direct kinase activity to these sites. The present study used the aforementioned genetic mouse models without residual CaMKII peptides but could not confirm a critical role of CaMKII after acute myocardial I/R injury. This conclusion based on three pieces of evidence: First, there was no difference in infarct sizes after 30 or 60 min of ischemia and subsequent 24 h of reperfusion. Second, neither apoptotic nor necrotic markers were significantly different. Third, we confirmed these data in four different CaMKII loss-of-function models: (i) KO of the major cardiac CaMKII isoform δ, (ii) functional KO of the major cardiac CaMKII splice variants δB or δC, (iii) cardiomyocyte-specific DKO, to address the redundant roles of CaMKIIδ and γ, and (iv) induced cardiomyocyte-specific DKO, to reduce compensatory mechanisms. Surprisingly, but based on these extensive studies, we must conclude that CaMKII—at least in the clinically relevant setting that was used in this study—is not critical for infarct size determination after acute myocardial ischemia/reperfusion injury in mice. Potential arrhythmogenic effects of CaMKII were not directly investigated in the present study. However, we did not observe any differences with regard to acute mortality of the operated animals, allowing us to assume that possible I/R-related arrhythmias were not CaMKII-mediated.

### CaMKII DKO alters leukocyte infiltration kinetics after I/R injury

Despite similar infarct sizes (based on hsTnT levels 24 h after ischemia) in FFFF and DKO mice, cardiac dysfunction failed to progress 3 weeks after I/R injury in DKO animals. The protection against cardiac dysfunction was associated with reduced scar formation. Thus, we searched for possible underlying mechanisms. Within the early phase after I/R injury between day 1 and 5, inflammatory signals recruit myeloid cells (neutrophils, monocytes/macrophages) to the infarct zone, where different populations of these leukocytes are responsible for clearance of dead cardiomyocytes and cell debris (Leuschner*et al*, 2012). In addition, formation of granulation tissue including promotion of new blood supply marks the beginning of post-infarct remodeling (Ertl & Frantz, 2005; Frangogiannis, 2008). It has been shown that distinct monocyte subsets contribute in specific ways to myocardial ischemic injury. Two distinct phases of monocyte participation after MI have been proposed: (I) Ly-6C hi monocytes have been shown to dominate the early phase and exhibit phagocytic, proteolytic, and inflammatory functions, while (II) Ly-6C lo monocytes dominate the later phase by expressing vascular endothelial growth factor and promoting healing via myofibroblast accumulation, angiogenesis, and deposition of collagen (Nahrendorf*et al*, 2007). In the present study, we used CD45 as a pan leukocyte marker to investigate general differences in leukocyte infiltration kinetics in CaMKII DKO mice. While at day 1 after I/R injury, no difference in inflammatory leukocyte response could be seen, we found altered leukocyte infiltration at day 5 after I/R injury. Considering different phases of post-I/R inflammatory processes and distinct functional differences of inflammatory cells at specific time points in post-I/R remodeling (Nahrendorf*et al*, 2007), we postulated that CaMKII might be a key regulator of the later inflammatory response after I/R—rather than an early regulator of cell death and infarct size determination, as evidenced by our acute I/R experiments (Figs1, 2 and 4, Supplementary Figs S2 and S7).

### How does CaMKII regulate cardiac inflammation?

Several previous studies reported protective effects of CaMKII inhibition in the chronic process of pathological remodeling after myocardial infarction or pathological pressure overload, which are both characterized with the activation of inflammatory pathways (Zhang*et al*, 2005; Backs*et al*, 2009; Ling*et al*, 2009; Yoo*et al*, 2009). Based on the conditional cardiomyocyte-specific knockout strategy used in this study, we can rule out that CaMKII mediates the inflammatory response by acting in inflammatory cells, in cardiac fibroblasts or other non-myocyte cells because CaMKII was specifically deleted in cardiomyoctes. Thus, we investigated whether CaMKII may signal via chemoattractant signals to leukocytes. In cardiomyocytes, CaMKII was shown to tightly regulate epigenetic processes controlled by histone deacetylases (HDACs) leading to transcriptional activation of gene programs that drive pathological remodeling (Anderson*et al*, 2011). In particular, CaMKII regulates HDAC4 and indirectly HDAC5, resulting in cytosolic accumulation of these epigenetic enzymes (Backs*et al*, 2006, 2009). As a consequence, gene programs that are controlled by the transcription factors MEF2 and SRF are activated (Kreusser & Backs, 2014; Lehmann*et al*, 2014). Cardiomyocyte-specific overexpression of these transcription factors leads to pathological remodeling (Zhang*et al*, 2001; Kim*et al*, 2008). However, leukocyte infiltration was not studied in detail in these animal models, but MEF2 was recently identified to be a critical part in inflammatory pathways in*Drosophila* (Clark*et al*, 2013). Moreover, it has been recently described that the CaMKII/HDAC4-HDAC5/MEF2-pathway (Backs*et al*, 2008) regulates CCL2 in vascular smooth muscle cells (Ginnan*et al*, 2012). Here, we show for the first time that gene expression of CCL2 and CCL3 is induced by CaMKII in cardiomyocytes and that CaMKII is required for hypoxia-induced CCL3 expression in cardiomyocytes. Both CCL2 and CCL3 have been implicated in cardiac pathologies: CCL2 (MCP-1) is a well-known chemokine involved in recruitment of leukocytes and regulated after myocardial infarction (Birdsall*et al*, 1997). CCL3 has been described to be upregulated after myocardial infarction, autoimmune myocarditis, and Chagas disease due to trypanosoma cruzi infection (Goser*et al*, 2005; Machado*et al*, 2008; Alves*et al*, 2012). However, the upstream signaling pathways of CCL3 under these conditions were incompletely understood. We now demonstrate that CaMKII is sufficient and required for CCL3 expression in cardiomyocytes.

### Clinical implications

We conclude that other than reported before CaMKII is not critical for cardiomyocyte survival and infarct size determination in the first 24 h after myocardial infarction. However, at later time points, combined deletion of the two cardiac CaMKII genes slows down the process of I/R-mediated post-infarct remodeling and cardiac dysfunction, possibly by attenuating pro-inflammatory chemoattractant signaling molecules such as CCL3. Therefore, CaMKII inhibition might become a promising anti-inflammatory therapeutic strategy in patients during post-infarct remodeling after myocardial infarction and revascularization. Moreover, given the role of CCL3 in autoimmune myocarditis and Chagas disease, CaMKII might also be a promising drug target in these disease entities.

## Materials and Methods

### Animals

CaMKIIδ and CaMKIIγ KO mice were described previously (Backs*et al*, 2009, 2010). CaMKIIδ/CaMKIIγ double knockout (DKO) mice were created by crossbreeding double-floxed animals (CaMKIIγ^loxP/loxP^; CaMKIIδ^loxP/loxP^, FFFF) with mice expressing Cre-recombinase under the control of a cardiomyocyte-specific α-MHC-promoter (Agah*et al*, 1997). Inducible CaMKII double KO mice (iDKO) were created by Cre-recombinase that was expressed under the control of a CMV-enhanced 260-bp myosin light chain (MLC260) promotor and delivered to the heart via cardiotrophic AAV9 (AAV9-MLC260-Cre) (Geisler*et al*, 2011). FFFF control mice received AAV9-MLC260-Luc (luciferase expression). Briefly, 9 × 10^11^vg of AAV9-MLC260-Cre or AAV9-MLC260-Luc were injected intravenously into tail veins of FFFF mice. Subsequent I/R experiments were performed at 12–13 weeks after i.v. injection. CaMKIIδ KO, DKO, and CaMKIIδ KO mice expressing CaMKIIδ splice variants δB and δC were subjected to surgery at the age of 10–15 weeks. Mice expressing AAV-Cre and AAV-Luc were operated at 16–18 weeks of age. All animals were fed a standard diet and water and were maintained on a 12-h light and dark cycle at a room temperature of 22 ± 2°C. All experimental procedures were reviewed and approved by the Institutional Animal Care and Use Committee at the Regierungspräsidium Karlsruhe, Germany.

Please see detailed Supplementary Methods providing methodological summary of*in vivo* I/R, high-sensitive cardiac Troponin T measurements, chemical reagents, plasmids and generation of AAV vectors, mitochondrial isolation, Western blot analysis, RNA microarray, RT–PCR, histology, TdT-mediated dUTP nick end labeling (TUNEL), caspase-3/7 activity measurements, transthoracic echocardiography, flow cytometry, primary cardiomyocyte cell culture experiments, and P-V loop analysis.

**The paper explained**

**Problem**

CaM Kinase II (CaMKII), a serine/threonine protein kinase with a broad spectrum of substrates, was suggested to mediate ischemic myocardial injury and adverse cardiac remodeling. Recent studies investigated the role of CaMKII in myocardial infarction and ischemia/reperfusion injury (I/R), mostly using inhibitory peptides and pharmacologic agents. However, the specific functions of the cardiac CaMKII genes and splice variants in I/R remained unclear.

**Results**

Here, we show that CaMKII is critically involved in the process of post-infarct remodeling, but—other than reported before—not in the immediate mechanisms that regulate acute damage after I/R injury. Using flow cytometry and gene profiling, we found that CaMKII mediates altered leukocyte infiltration kinetics and pro-inflammatory chemoattractant signaling, indicating that CaMKII regulates pathological post-infarct remodeling via inflammatory pathways rather than via early induction of cell death. This was confirmed by the use of different new genetic CaMKII KO mouse models: While (i) neither the single genetic deletion of the CaMKIIδ isoform, nor (ii) a gene replacement strategy with adeno-associated virus-mediated expression of the CaMKII splice variants δB and δC, nor (iii) a cardiomyocyte-specific CaMKIIδ/γ double knockout (DKO) nor (iv) inducible DKO did reveal differences in infarct size or cell death in acute I/R injury, 5 weeks after I/R injury, DKO mice were protected against extensive scar formation and cardiac dysfunction, as assessed by trichrome staining, transthoracic echocardiography and pressure-volume loop measurements.

**Impact**

CaMKII inhibition might become a promising therapeutic anti-inflammatory strategy in patients during post-infarct remodeling after myocardial infarction and revascularization. However, other than hypothesized, CaMKII inhibition seems not to prevent acute cardiac damage 1 day after ischemia.

## Statistics

Data are presented as mean ± SEM and were analyzed by Student's*t*-test between two groups or by ANOVA with Bonferroni's multiple comparison test when 3 or more groups were compared. A*P*-value < 0.05 was considered statistically significant.
