# Supplementary material for: CaM Kinase II mediates maladaptive post-infarct remodeling and pro-inflammatory chemoattractant signaling but not acute myocardial ischemia/reperfusion injury
Source: EMBO Mol Med. 2014 Sep 5;6(10):1231–45. doi: 10.15252/emmm.201403848 (PMC4287929; doi:10.15252/emmm.201403848)
Supplement: Supplementary file 1 [file emmm0006-1231-sd1.pdf]

# Supplementary Information

**Supplemental Materials and Methods**________________________________________ **1**

**Supplemental Figures**_______________________________________________________ **4**

**Supporting Information References**_________________________________________ **17**

# Supplemental Materials and Methods

**Ischemia/Reperfusion.** Mice were anesthetized and subjected to myocardial I/R. Briefly, after initiation of general anesthesia with Propofol 1% (0.002 ml/g bodyweight, retroorbital injection) and isoflurane anesthetic gas, the mice were intubated, ventilated with a rodent ventilator (MiniVent Type 845, Harvard Apparatus, Holliston, MA) and a left thoracotomy was performed. Anesthesia was maintained with isoflurane 2.0 %. The left anterior descending (LAD) coronary artery was ligated with an 8-0 suture, tied on top of a plastic tube, 0.9 mm in diameter, approximately 1 mm below the edge of the left atrial appendage. Successful ligation of the artery was confirmed by blanching of the left ventricular (LV) myocardium. After 30 or 60 minutes of ischemia, reperfusion was established by pulling out the occluding tube. 24 hours post-surgery, the LAD was re-occluded and 2 % Evans Blue injected into the beating LV of terminally anesthetized mice to delineate the ischemic area. Hearts were harvested and 7-8 parallel transverse sections (thickness 1 mm) were obtained. Infarct area was determined by incubation with 1 % triphenyltetrazolium chloride (TTC). Each heart was digitally recorded with a microscope and a digital camera (Canon Ixus, Canon Inc., Tokyo, Japan). All stained transverse sections were photographed from top and bottom. The total heart volume, ischemic area (area at risk) and infarct area were determined with computer-assisted planimetry software (Image J, http://rsbweb.nih.gov/ij/). All data were analyzed by a single observer blinded to mouse genotypes.

**High Sensitive Cardiac Troponin T Measurements.** Retroorbital blood samples were taken from isoflurane anesthetized mice using hematocrit capillaries. Fresh blood samples were centrifuged to obtain serum. Further analysis was performed using an automated Cobas Troponin T hs STAT Elecsys (Roche).

**RNA isolation.** Total RNA was isolated from ventricular tissue or from cultured cardiomyocytes using TRIzol (Invitrogen, Germany). Total RNA was digested with DNase, and cDNA synthesis from 500 ng of RNA was carried out using a SuperScript first-strand synthesis system for RT-PCR (Invitrogen).

**Quantitative real-time PCR (qPCR).** qPCR was performed with Universal ProbeLibrary (Roche) by using TaqMan Universal PCR Mastermix (Applied Biosystems) and detection on a 7500 Fast Cycler (Applied Biosystems). Primers and probes were: For rat: PPBP 5´-gcttcagactcagacctacatcc-3´ and 5´-aaatggctcgttgttatcagg-3´; CXCR2 5´-cagagacttgggagccactc-3´ and 5´-gctgaaattatccaccctgatt-3`; CCR1 5-´gactctcaaaggcccagaaa-3` and 5´-ggaactggtcaggaacaatagc-3`; CXCL3 5`-ggctcctcaatgctgcac-3` and 5`-ggccacaacagtccctga-3`; CCL2 5`-cgtgctgtctcagccagat-3` and 5`-ggatcatcttgccagtgaatg-3`; CCL6 5`-atccttgtggccgtcctt-3` and 5`-agggacgatcttcttttaccg-3`; FGR 5`-tgttcccagcaactatgtgg-3` and 5`-tgctgatctttccgaagtacc-3`; HCK 5`-agccactgccaaaactcatt-3` and 5`-tgctcaatgaaagccatgc-3`; VAV1 5`-cgctccaagacagacaggta-3` and 5`-gcgcttacagatgagcagtg-3`; PIK3CD 5`-tggctggtggtccttctc-3` and 5`-gggcatcctgctgtgttact-3`; GAPDH 5`-tgggaagctggtcatcaac-3` and 5`-catcaccccatttgatcttga-3`. For mouse: PPBP 5`-gcccacttcataacctccag-3` and 5`-gggtccatgccatcagatt-3`; CXCR2 5`-caggaccaggaatgggagta-3` and 5`-tcccctccaaatatccccta-3`; CCR1 5`-tggacaaaatactctggaaacaca-3` and 5`-tgtgaaatctgaaatctccatcc-3`; CXCL3 5`-ggctcctcaatgctgcac-3` and 5`-ggccacaacagtccctga-3`; CCL2 5`-catccacgtgttggctca-3` and 5`-gatcatcttgctggtgaatgagt-3`; CCL6 5`-tctttatccttgtggctgtcc-3` and 5`-tggagggttatagcgacgat-3`; FGR 5`-ttccatccaggctgaagagt-3` and 5`-ctgggggttaccagaggac-3`; HCK 5`-acggagttcatggccaaa-3` and 5`-gcttgctgccttcttcactc-3`; VAV1 5`-ccagcaagcacttggatca-3` and 5`-tcggccattgttagctcttt-3`; GAPDH 5`-ccttgagatcaacacgtaccag-3` and 5`-cgcctgtacactccaccac-3`.

**Microarray analysis.** Gene expression profiling was performed with arrays of mouse mogene20-st-v1-type from Affymetrix. First, Biotinylated antisense cRNA was prepared according to the Affymetrix standard labelling protocol. Then, the hybridization on the chip was performed on a GeneChip Hybridization oven 640, afterwards dyed in the GeneChip Fluidics Station 450 and thereafter scanned with a GeneChip Scanner 3000. All of the equipment used was obtained from the Affymetrix-Company (Affymetrix, High Wycombe, UK). A Custom CDF Version 18 with Entrez based gene definitions was used for annotation of the arrays. The Raw fluorescence intensity values were normalized applying quantile normalization. Differential gene expression was analysed with an ANOVA, using a commercial software package (SAS JMP10 Genomics, version 6) from SAS (SAS Institute, Cary, NC, USA). A false positive rate of a=0.01 was taken as level of significance. Gene Set

Enrichment Analysis (GSEA), was used to determine whether defined lists or sets of genes exhibit a statistically significant bias in their distribution within a ranked gene list (see http://www.broadinstitute.org/gsea/for details (Subramanian et al, 2005). Pathways belonging to various cell functions, such as chemokine signalling or cytokine- cytokine receptor interaction were obtained from public external databases (KEGG, http://www.genome.jp/kegg/). The raw and normalized data are deposited in the Gene Expression Omnibus database (http://www.ncbi.nlm.nih.gov/geo/; accession No.: GSE58486).

**Mitochondria Isolation.** We isolated mitochondria from adult mouse hearts using an established method optimized for small tissue samples as described by others before.(Mela & Seitz, 1979; Wei et al, 2011) Briefly, the LV myocardium was manually homogenized for 6 min in the presence of protease inhibitor (complete mini EDTA free, Hoffmann-La Roche and DTT 2mM, Sigma) in ice-cold isotonic isolation solution (IS; 75 mM sucrose, 225 mM mannitol, 2mM HEPES and 1 mM EGTA, pH 7.4). The homogenate was centrifuged at low speed (480g, 5 min, 4°C) and the supernatant was further centrifuged (7700g, 10 min) to obtain the mitochondrial pellet. The supernatant was frozen in liquid nitrogen and used as cytoplasmic fraction for further analysis, the pellet containing mitochondria was washed twice in IS, once in IS without EGTA, centrifuged (7700g, 5min), and finally resuspended in IS without EGTA. Mitochondrial protein concentrations were determined using NanoDrop 2000 spectrophotometer (Thermo Scientific). Furthermore, purity of mitochondrial fraction was confirmed by Western Blot analysis using an Antibody to detect Cox IV (Cell Signaling, #11967).

**Flow cytometry.** To prepare single-cell suspensions from infarct tissue, hearts were harvested; minced with fine scissors; placed into a cocktail of collagenase I, collagenase XI, DNase I, and hyaluronidase (Sigma-Aldrich); and shaken at 37°C for 1 hour, as described previously(Leuschner et al, 2011). Cells were then triturated through nylon mesh and centrifuged (15 min, 500 g, 4°C). Cell suspensions were incubated with mAbs against CD45 (BD Biosciences). Data were acquired on a FACSCanto (BD Biosciences) and analyzed with FlowJo v.8.5.2 (Tree Star, Inc.).

**Chemical Reagents, Plasmids and AAV vectors.** Evans Blue and TTC were purchased from Sigma-Aldrich, St. Louis, MO. 4',6-diamidino-2-phenylindole, dihydrochloride (DAPI) and DNase I were obtained from Invitrogen Carlsbad, CA. Epitope-tagged derivates of the CaMKIIδ splice variants B and C containing amino-terminal Myc tags were described before(Backs et al, 2006) and subcloned into $pUFCMV_{enh}$/MLC0.26-Luc(Schinkel et al, 2012). AAV9-MLC260-CaMKIIδB, AAV9-MLC260-CaMKIIδC, AAV9-MLC260-Cre and AAV9-MLC260-Luc (as a control) were produced as described previously (Goehringer et al, 2009).

**Western Blot Analysis.** Cardiac extracts were prepared as described previously (Backs et al, 2009), and Western blot analysis was carried out as described in an earlier publication (Backs et al, 2006). Antibodies for immunoblotting were used as follows: rabbit anti-Myc (Santa Cruz Biotechnology), mouse anti-phospholamban (anti-PLN) (Upstate), rabbit anti-phospho-PLN (Thr-17) (Upstate), rabbit anti-phospho-PLN (Ser-16), mouse anti-GAPDH (Millipore), mouse anti-CaMKII (BD Bioscience) and rabbit anti-CaMKII (Millipore).

**Histology, TdT-mediated dUTP nick end labeling (TUNEL).** Hearts were harvested 24 hours or 35 days after surgery. 1-mm middle transverse sections (at 3 mm above the heart apex) were fixed in 4% formaldehyde, embedded in paraffin and stained using Roche's "*In situ* Cell Death Detection Kit, Fluorescein". All processing and staining was performed according to manufacturer's instructions. Negative control samples were stained according to the same protocol with the exception of TUNEL enzyme solution. Pretreatment with DNase I was used to obtain positive controls. Additional nucleus staining with 4',6-diamidino-2-phenylindole (DAPI) was performed using 25µl of DAPI, diluted 1:10,000 in PBS, per tissue sample. Samples were incubated for 15 minutes at room temperature and rinsed twice with PBS. Photographs were acquired with an Olympus SZH zoom stereo dissection scope with an Optronics DEI-750 CCD digital camera. An average of 400–500 LV nuclei per animal was analyzed with Image J. All data were analyzed by a single observer blinded to mouse genotypes. Sections obtained at 35 days after surgery were fixed in 4% formaldehyde for 24 hours at 4°C and then embedded in paraffin. Staining with Masson's trichrome was performed following a standard protocol as described before (Backs et al, 2009). Stained 3-µm sections were used for light microscopy and morphometric evaluation. Scar size quantification was performed with Image J by a single observer blinded to mouse genotypes.

**Caspase 3/7 Activity Measurements.** The Caspase-Glo 3/7 Assay (Promega Corporation) was used to measure caspase-3 and -7 activities. Heart lysates were mixed with reaction solution at equal volumes in a 96-well plate. Luminescence was detected after 60 minutes of incubation at room temperature. Left ventricular values were normalized to Caspase-3 and -7 activities in septal myocardium.

**Transthoracic Echocardiography.** Cardiac function was evaluated by 2D echocardiography on conscious mice as described before.(Backs et al, 2009) M mode tracings were used to measure left ventricular internal diameter as the largest anteroposterior diameter in either diastole or systole. All data were analyzed by a single observer blinded to mouse genotypes. Left ventricular fractional shortening was calculated.

**P-V loop analyses.** Closed chest P-V (pressure-volume) measurements were performed by LV catheterization as previously described.(Pacher et al, 2008; Shioura et al, 2007) Introduction of anaesthesia and tracheal intubation were performed as described above. Briefly, the right carotid artery was exposed and a 1.4 F pressure-conductance catheter with 4.5 mm electrode spacing (FTM-1212B-4518; Scisense, London, ON, Canada) was retrogradely advanced into the LV. After optimization of catheter tip position, anaesthesia was reduced to 0.5 % Isoflurane to provide physiological hemodynamic readings. Steady-state P-V measurements were obtained and registered using the MPVS-300 (Millar Instruments, Houston, TX) and Chart 5.0 with PVAN 3.5 analysis software (ADInstruments, Colorado Springs, CO). Finally, mice were euthanized through 5 % Isoflurane inhalation and blood samples were collected by terminal bleeding.

**Adenovirus production.** Adenoviruses harboring Myc-CaMKII-T287D (CaMKII) were generated according to the manufacturer's instructions (ViraPower Adenoviral Expression System; Invitrogen). Nuclear factor of activated T-cells (NFAT)-GFP adenovirus was obtained from Seven Hills Bioreagents (Cincinnati, USA). After generation, the adenoviruses were amplified, purified with the Adeno-X Purification Kit (BD) and its infectious units per µl were determined with the Adeno-X Rapid Titer Kit (BD).

**Culture of neonatal rat ventricular cardiomyocytes (NRVMs).** NRVMs were isolated from 1 to 2-day old Wistar rats as previously described (Backs et al, 2006). After isolation, NRVMs were maintained in DMEM/199 medium (4:1) with 10% FBS, 2 mM l-glutamine, and penicillinstreptomycin. NRVMs were infected 72 h after plating, grown 24 h later in serum-free media for another 8 h. For hypoxia experiments, NRVMs were incubated in hypoxic (1,5% $O_2$) chambers for 1 h followed by 6 or 24 h of normoxia (ambient air). 24 h of normoxia served as control condition.

**Culture of adult mouse ventricular myocytes (AMVMs).** For AMVM isolation DKO mice and their Cre-negative littermates, respectively, were anesthetized with isoflurane and hearts were excised. Explanted hearts were retrogradely perfused and digested as described (Kohlhaas et al, 2010). Cells were plated onto regular cell culture plates with the glass bottoms treated with laminin. After 1 h in the fresh complete medium, hypoxia experiments were started. AMVMs were incubated in hypoxic (1,5% $O_2$) chambers for 1 h followed by 8 h of normoxia (ambient air). 9 h of normoxia served as control condition.

**Determination of supernatant CCL2 and CCL3.** Concentration of CCL2 and CCL3 were measured in cell culture supernatants from NRVMs by enzyme-linked immunosorbent assay according to the manufacturer´s instructions (R&D mouse/rat CCL2/JE/MCP-1 quantikine ELISA kit; Abnova rat CCL3 ELISA kit).

## Supplemental Figures

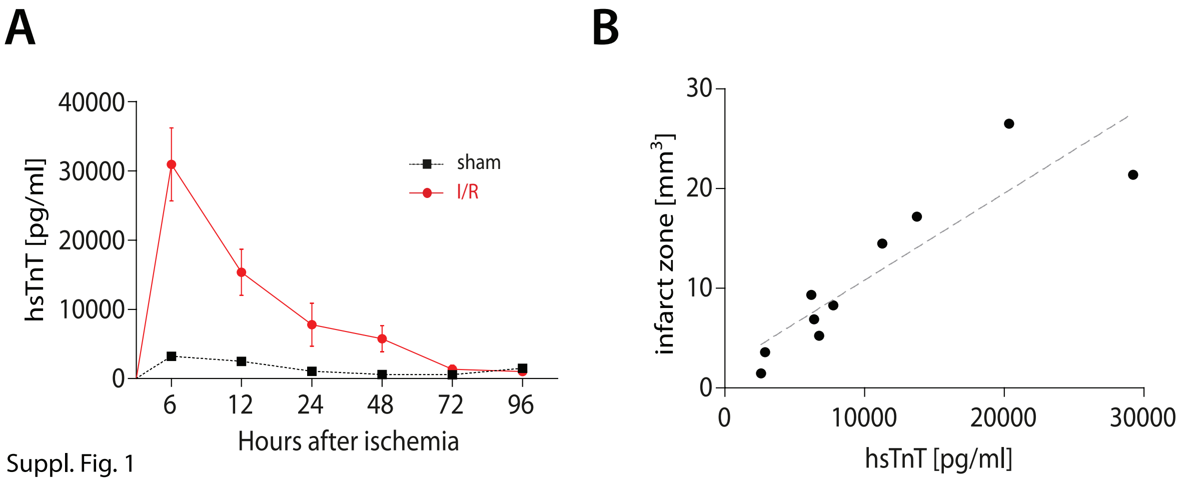


Suppl. Fig. 1

**Suppl. Fig 1. High sensitive serum Troponin T (hsTnT) is a reliable marker to detect myocardial damage in murine I/R injury. (A)** High sensitive serum Troponin T was measured in C57BL/6 (WT) mice at various time points after I/R surgery or sham operation as depicted in graph. N ≥ 5 in each group. **(B)** Correlation of hsTnT values with infarct zone area (IZ). WT mice were subjected to I/R surgery. 24 hours after I/R injury, hearts were harvested and underwent Evans Blue and TTC staining. IZ was calculated planimetrically. Dashed line: linear regression, Pearson $r = 0.89$ with $p < 0.001$.

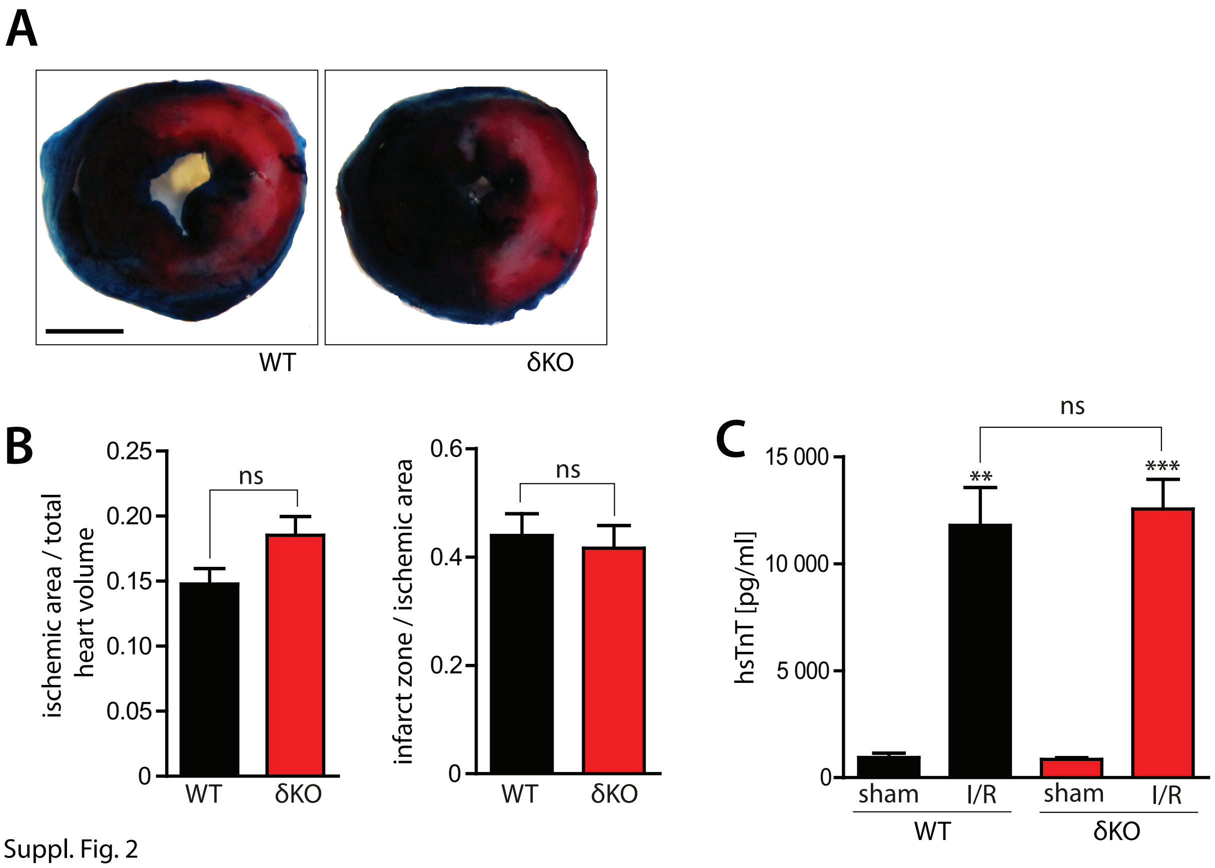


**Suppl. Fig 2. 30 minutes of ischemia in WT vs. CaMKIIδ KO mice.** I/R surgery was performed in CaMKIIδ$^{-/-}$ C57BL/6 (KO) and wild-type C57BL/6 (WT) mice. **(A)** Transverse heart sections were obtained from WT and KO mice. All sections were stained with Evans Blue and TTC. Representative images are shown. Scale bar: 2 mm. **(B)** Quantitative measurement of ischemic area / total heart volume ratio and infarct zone / ischemic area ratio was performed. Planimetrically and volumetrically calculated infarct sizes show no significant differences. N = 19 WT animals, 20 KO animals operated and analyzed per group. Infarct zone / ischemic area: ns, p = 0.69. Ischemic area / total heart volume: ns, p = 0.54. **(F)** High sensitive serum Troponin T (hsTnT) was measured 24 hours after myocardial I/R injury. N = 5 WT sham animals, 10 KO sham animals, 19 WT I/R animals, 20 KO I/R animals. ns, p = 0.73; **, p = 0.006; ***, p = 0.0001. All data are expressed as mean ± SEM. Unpaired Student's t-test (B) and One-way ANOVA (C) were used to compare groups. ns = non-significant.

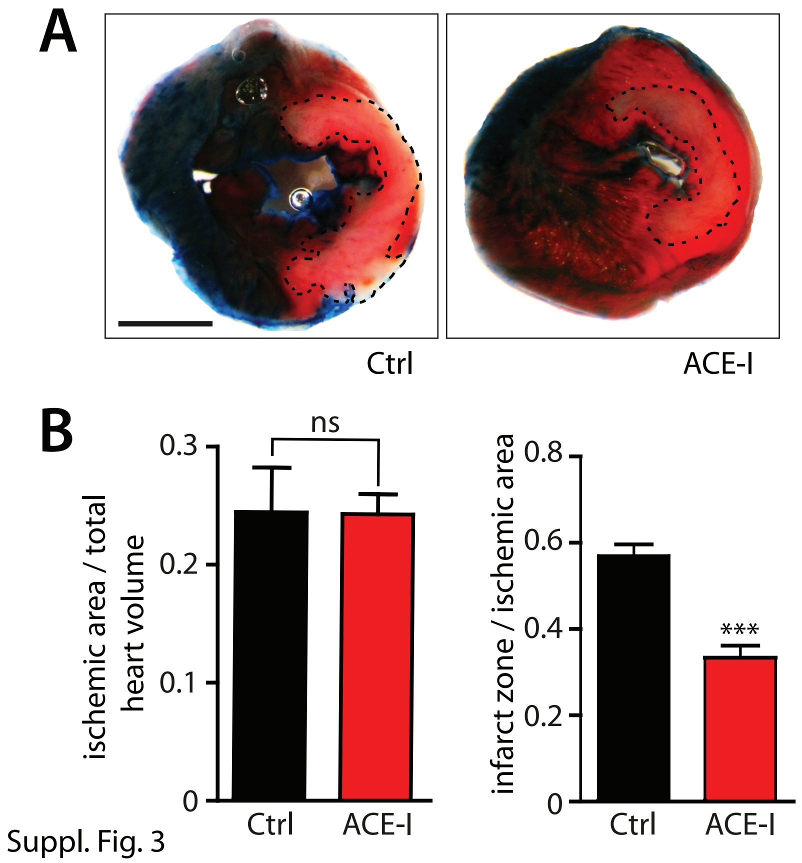


**Suppl. Fig 3. Reduced infarct size in Enalapril pretreated WT animals after 60 minutes of ischemia and 24 hours of reperfusion.** I/R surgery was performed in C57BL/6 WT mice without pretreatment (Ctrl) and with pretreatment with the ACE-inhibitor Enalapril (ACE-I). **(A)** Transverse heart sections were obtained from Ctrl and ACE-I mice 24 hours after I/R surgery. All sections were stained with Evans Blue and TTC. Representative images are shown. Scale bar: 2 mm. Dotted line: whitened infarct zone within ischemic area. **(B)** Quantitative measurement of ischemic area / total heart volume ratio and infarct zone / ischemic area ratio was performed. Planimetrically and volumetrically calculated infarct sizes were significantly smaller in ACE-I mice. N = 5 animals operated and analyzed per group. ns, $p = 0.98$; ***, $p = 0.0003$. All data are expressed as mean ± SEM. Unpaired Student's t-test was used to compare groups. ns = non-significant.

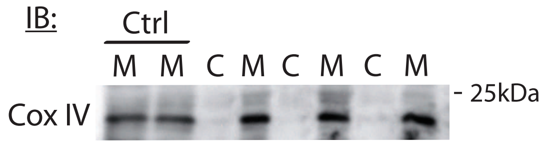


Suppl. Fig. 4

**Suppl. Fig 4.** Purity of cytoplasmic and mitochondrial fraction was validated using Western Blot analysis with an antibody directed against mitochondrial Cox IV (predicted molecular weight approximately 19 kDa). Shown is a representative blot of 3 samples. Two mitochondrial lysates from independent experiments served as control in each individual experiment (Ctrl). C = cytoplasmic, M = mitochondrial fraction of each sample.

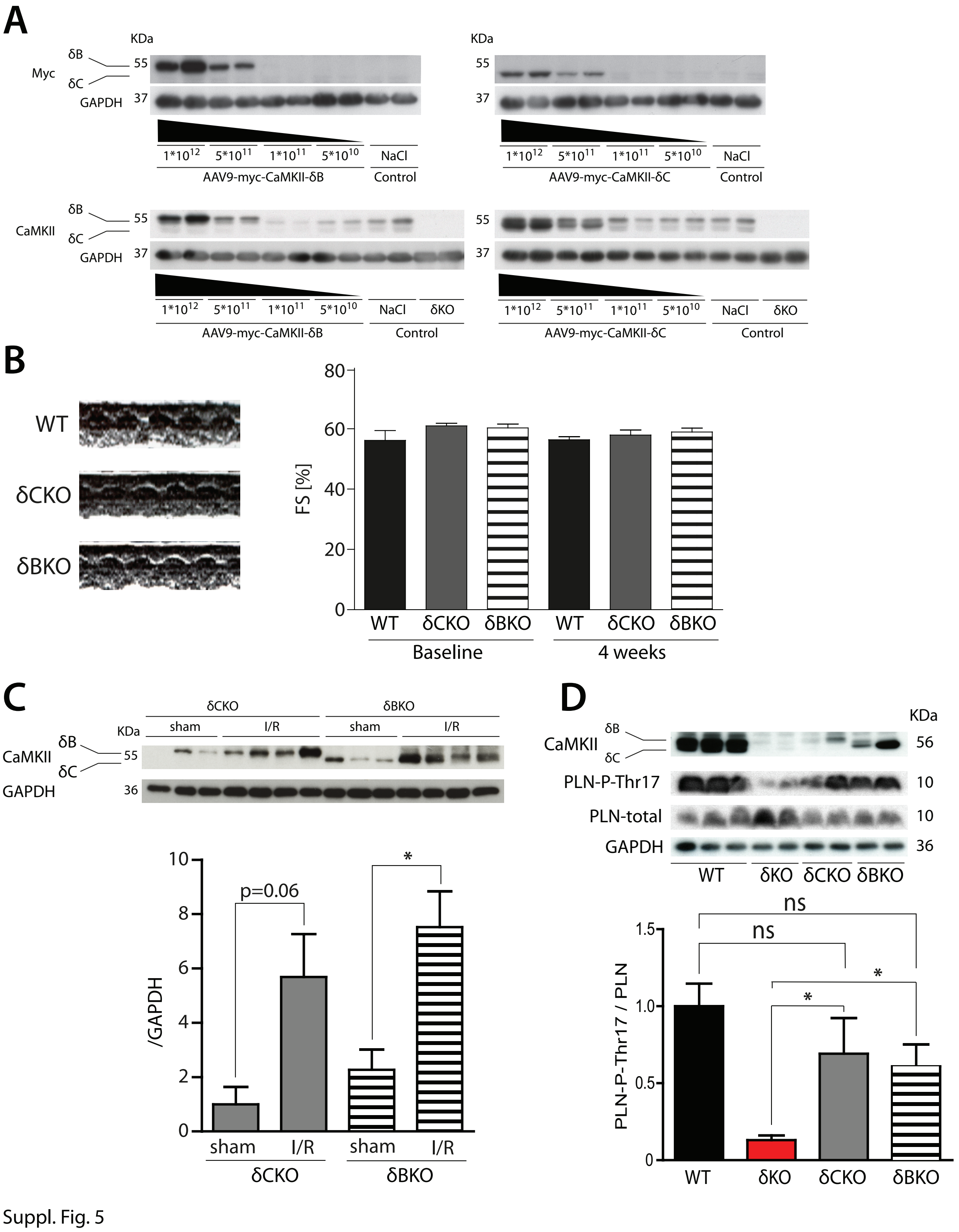


**Suppl. Fig 5. A reliable gene transfer approach to express CaMKIIδ splice variants. (A)** C57BL/6 wild-type mice received a single AAV9 tail vein injection of decreasing AAV9 titers as indicated. NaCl injection served as control. Hearts were harvested four weeks later. (*Upper*) Western blot analysis of viral CaMKIIδ splice variant expression using an antibody against the myc-tag and GAPDH as loading control. (*Lower*) Western blot analysis using an antibody against CaMKII, detecting both CaMKIIδ splice variants. Upper part of double band represents CaMKIIδB splice variant. GAPDH served as loading control. *δKO* = homogenates obtained from CaMKIIδ KO mice. **(B)** Transthoracic echocardiography was performed to assess cardiac function at baseline and four weeks after AAV9 injection. Bar graphs show left ventricular fractional shortening (FS). FS did not

differ significantly between the groups. N = 5 animals in WT group, 15 animals in δCKO group, 20 animals in δBKO group. Data are mean ± SEM. One-way ANOVA was used to compare groups. **(C)** Western blot analysis of CaMKIIδ splice variant expression, 24 hours after I/R injury in AAV9-CaMKIIδB/C-expressing CaMKIIδ-KO mice. One-way ANOVA was used to compare groups. *, p = 0.03. **(D)** Western blot analysis of CaMKIIδ splice variant expression in unstressed animals: AAV9-CaMKIIδB/C-expressing CaMKIIδ-KO mice, WT mice and CaMKIIδ-KO mice. N = 6 animals per group in quantification analyses (two independent Western Blot experiments). All data are expressed as mean ± SEM. One-way ANOVA was used to compare groups. ns = non-significant; ns, p = 0.27 in WT vs. δCKO; ns, p = 0.11 in WT vs. δBKO; *, p = 0.04 in δKO vs. δCKO; *, p = 0.02 in δKO vs. δBKO.

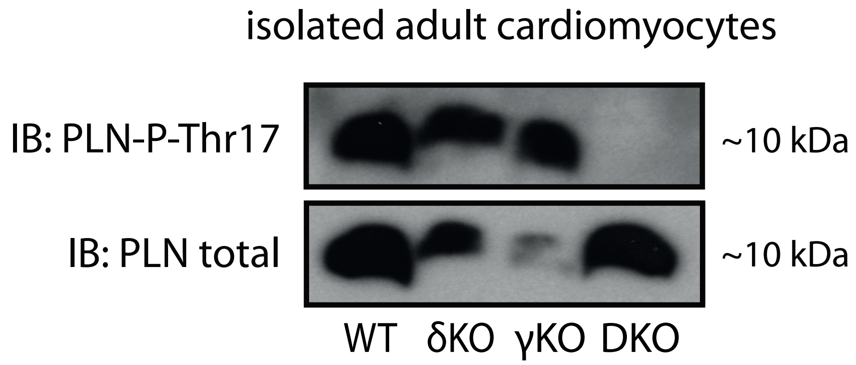


Suppl. Fig. 6

**Suppl. Fig 6. PLN-P-Thr17 phosphorylation in different CaMKII KO mouse models.**
Western Blots showing PLN phosphorylation as indicator for CaMKII activity in isolated adult cardiomyocytes from wilde-type mice, CaMKII gamma KO, delta KO and CaMKII delta/gamma double KO mice.

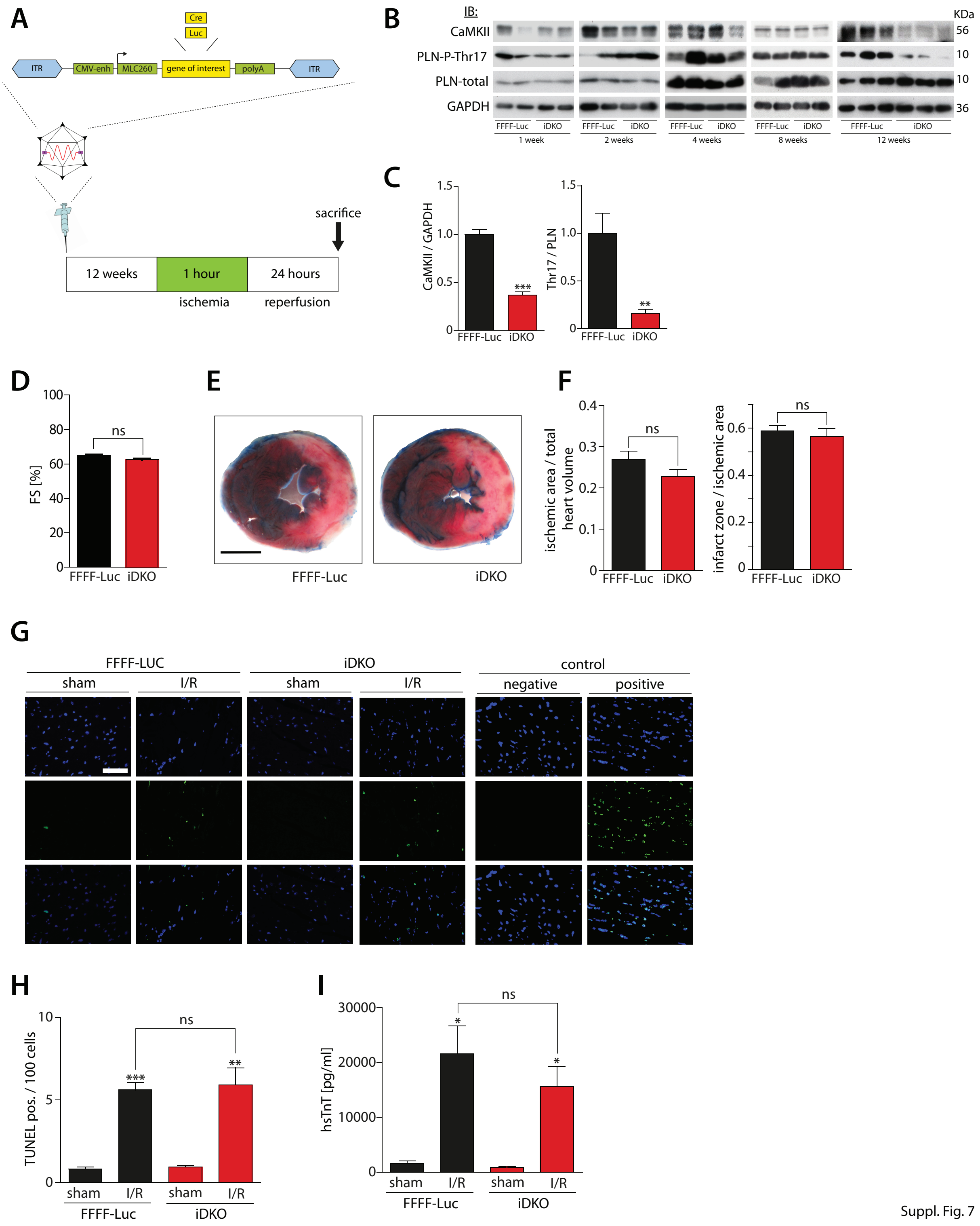


**Suppl. Fig 7. I/R in an inducible CaMKII KO model using AAV9. (A)** AAV9 vectors were produced to express Cre-recombinase (Cre) in CaMKIIγ$^{loxP/loxP}$; CaMKIIδ$^{loxP/loxP}$ (FFFF) mice, resulting in an induced DKO (iDKO). Viruses containing the Luciferase (Luc) gene served as control (FFFF-Luc). Following a single tail vein injection, murine hearts were sacrificed after one, two, four, eight and twelve weeks to quantify the gene take out using Western blot analysis. **(B)** Representative Western blots and quantification showing significant reduction of cardiac CaMKII protein levels in iDKO versus FFFF-Luc 12 weeks after virus injection. At earlier time points (one, two, four and eight weeks after virus injection), gene deletion as detected by Western Blot was not sufficient. Total amounts of PLN and GAPDH served as loading controls. **(C)** Phosphorylated PLN at the CaMKII phosphorylation site Thr-17 was reduced by 84% in iDKO compared to FFFF-Luc control group. Shown are quantification bar graphs of 12 week time point. N = 7 in each group. **, $p = 0.001$; ***, $p < 0.0001$. **(D)** Transthoracic echocardiography was performed to assess cardiac function of AAV9-Cre and AAV9-Luc

expressing animals, twelve weeks after intravenous injection. N = 4 animals in FFFF-Luc group, 5 animals in iDKO group. ns, p = 0.68. **(E)** Twelve weeks after AAV9 injection, mice underwent I/R surgery. Transverse heart sections were obtained from FFFF-Luc and iDKO mice at 24 hours after myocardial I/R injury. Evans Blue and TTC staining was performed. Representative images are shown. Scale bar: 2 mm. **(F)** Planimetric and volumetric measurement of ischemic area / total heart volume ratio and infarct zone / ischemic area ratio was performed. No significant differences between the groups were found. Ischemic area / total heart volume: ns, p = 0.15. Infarct zone / ischemic area: ns, p = 0.59. **(G)** Representative photomicrographs of TUNEL-staining in FFFF-Luc and iDKO LV transverse sections after I/R or sham surgery. Positive: pretreatment with DNase I; negative: without TUNEL enzyme solution. Scale bar: 50 µm. (*upper*) DAPI, (*middle*) TUNEL, (*lower*) merge. **(H)** Bar graphs show quantification of TUNEL staining. N = 4 per group. ns, p = 0.78; **, p = 0.01; ***, p = 0.0003. **(I)** hsTnT was measured 24 hours after myocardial I/R injury. N = 4 animals per sham group, 7 animals per I/R group. ns, p = 0.36; *, p = 0.02. Data are mean ± SEM. Unpaired Student's t-test (C, D, F) and One-way ANOVA (H, I) were used to compare groups. ns = non-significant.

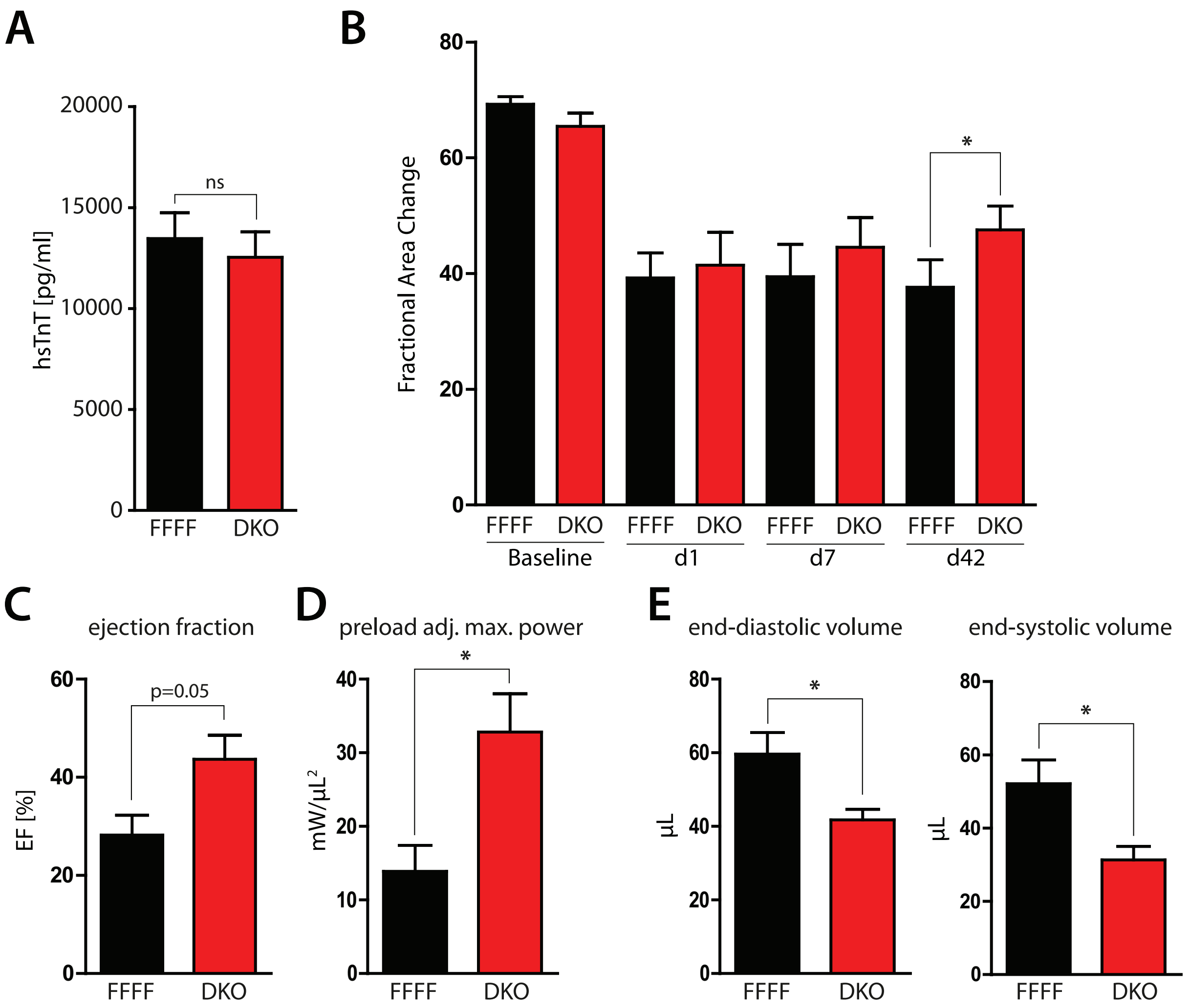


Suppl. Fig. 8

**Suppl. Fig. 8. Improved cardiac function in DKO mice six weeks after I/R surgery.** DKO and FFFF littermates were subjected to I/R with six weeks of follow-up. Echocardiography was performed at baseline, one day, seven days and six weeks post-surgery. In addition, at six weeks, invasive hemodynamic parameters were assessed. **(A)** hsTnT was measured 24 hours after myocardial I/R injury and revealed equal infarcts sizes in DKO and FFFF at this time point. N = 9 animals per group. ns, p = 0.61. **(B)** Transthoracic echocardiography revealed a significant difference in fractional area change (FAC) between FFFF and DKO six weeks after I/R surgery. N = 7 animals per group. *, p = 0.04. **(C-E)** Pressure-volume loop quantification data showing improved hemodynamic cardiac function in DKO as compared to FFFF controls with less cardiac dilation in DKO animals. N = 7 animals per group. *, p = 0.02 in D; *, p = 0.02 in E. Unpaired Student's t-test (A, C-E) and One-way ANOVA (B) were used to compare groups.

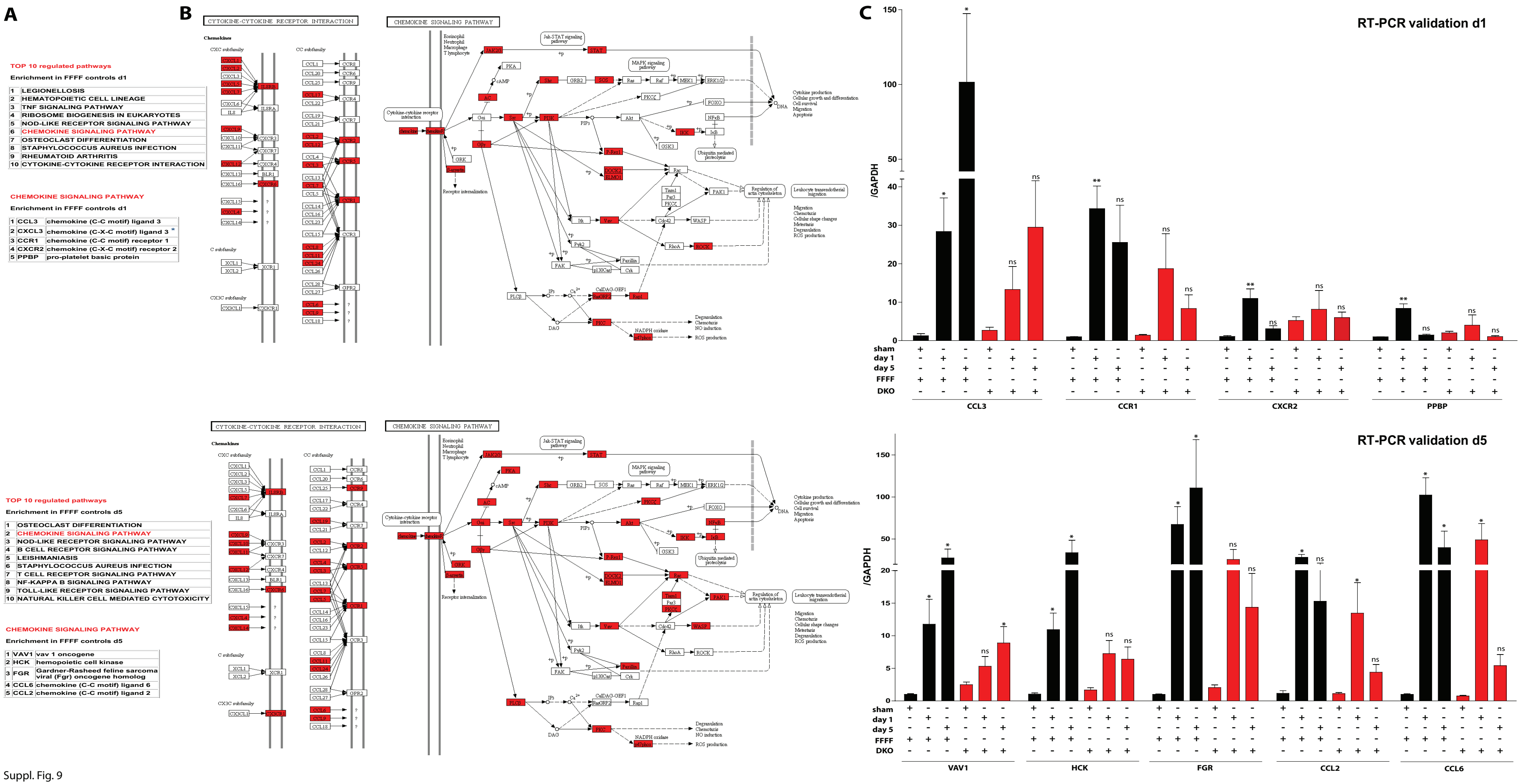


Suppl. Fig. 9

**Suppl. Fig. 9. Gene expression and pathway analyses reveal CaMKII-dependent pro-inflammatory genes regulated upon I/R injury. (A)** Pathways belonging to specific cell functions were obtained from public external databases (KEGG PATHWAY Database, http://www.genome.jp/kegg/) to perform pathway analyses. Top ten regulated pathways are shown for 1 and 5 day after I/R injury. Pathways listed are highly significantly up-regulated in FFFF control animals as compared to DKO mice. Next, "Chemokine Signaling Pathway" (marked red) was further analysed; the top 5 genes that were attenuated in DKO are shown in tables below. Blue asterisk: CXCL3 could not be detected in

validation experiments. **(B)** Graphical pathway visualization for Chemokine-Receptor Interaction Pathway and Chemokine Signaling Pathway. Regulated genes are marked red. **(C)** Bar graphs showing RT-PCR validation of top 5 regulated genes (except: CXCL3. This could not be detected in validation experiment) of Chemokine Signaling Pathway. N = 4 animals per group. CCL3: *, p = 0.02, sham vs. day 1 in FFFF; *, p = 0.01, sham vs. day 5 in FFFF; ns, p = 0.13, sham vs. day 1 in DKO; ns, p = 0.07, sham vs. day 5 in DKO; CCR1: **, p = 0.001, sham vs. day 1 in FFFF; ns, p = 0.05, sham vs. day 5 in FFFF; ns, p = 0.11, sham vs. day 1 in DKO; ns, p = 0.10, sham vs. day 5 in DKO; CXCR2: **, p = 0.001, sham vs. day 1 in FFFF; ns, p = 0.05, sham vs. day 5 in FFFF; ns, p = 0.28, sham vs. day 1 in DKO; ns, p = 0.68, sham vs. day 5 in DKO; PPBP: **, p = 0.001, sham vs. day 1 in FFFF; ns, p = 0.06, sham vs. day 5 in FFFF; ns, p = 0.49, sham vs. day 1 in DKO; ns, p = 0.09, sham vs. day 5 in DKO; VAV1: *, p = 0.03, sham vs. day 1 in FFFF; *, p = 0.04, sham vs. day 5 in FFFF; ns, p = 0.12, sham vs. day 1 in DKO; *, p = 0.04, sham vs. day 5 in DKO; HCK: *, p = 0.009, sham vs. day 1 in FFFF; *, p = 0.04, sham vs. day 5 in FFFF; ns, p = 0.05, sham vs. day 1 in DKO; ns, p = 0.05, sham vs. day 5 in DKO; FGR: *, p = 0.02, sham vs. day 1 in FFFF; *, p = 0.01, sham vs. day 5 in FFFF; ns, p = 0.09, sham vs. day 1 in DKO; ns, p = 0.06, sham vs. day 5 in DKO; CCL2: *, p = 0.003, sham vs. day 1 in FFFF; ns, p = 0.09, sham vs. day 5 in FFFF; *, p = 0.04, sham vs. day 1 in DKO; ns, p = 0.06, sham vs. day 5 in DKO; CCL6: *, p = 0.0002, sham vs. day 1 in FFFF; *, p = 0.04, sham vs. day 5 in FFFF; *, p = 0.04, sham vs. day 1 in DKO; ns, p = 0.05, sham vs. day 5 in DKO. Data are expressed as mean ± SEM. One-way ANOVA with Bonferroni's Multiple Comparison Test was used to compare groups. ns = non-significant.

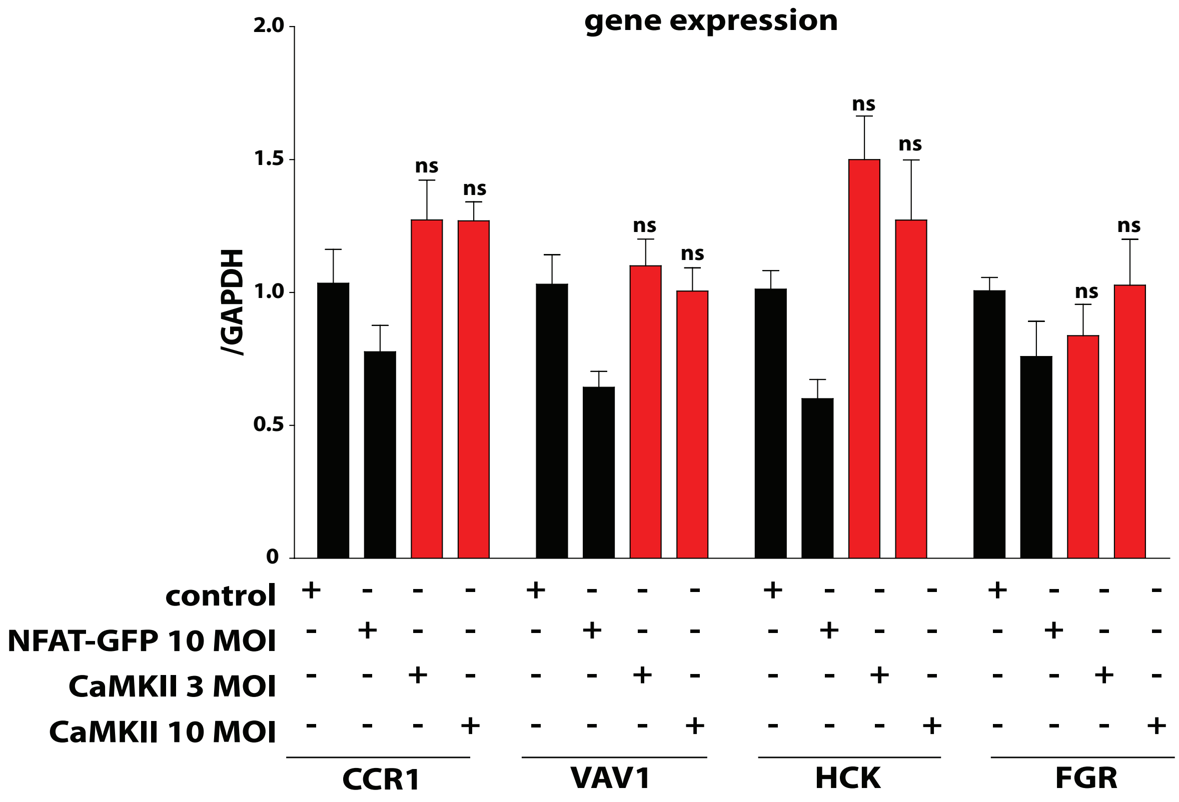


Suppl. Fig. 10

**Suppl. Fig. 10. CaMKII did not induce expression of intracellular components of inflammatory cells.** Gene expression of inflammatory genes in NRVMs after infection with active CaMKII (T287D), NFAT-GFP or control. Gene expression is normalized to GAPDH. N = 6 per group. CCR1: ns, p = 0.10 in 3 MOI; ns, p = 0.34 in 10 MOI; VAV1: ns, p = 0.65 in 3 MOI; ns, p = 0.86 in 10 MOI; HCK: ns, p = 0.05 in 3 MOI; ns, p = 0.30 in 10 MOI; FGR: ns, p = 0.22 in 3 MOI; ns, p = 0.91 in 10 MOI. Data are expressed as mean ± SEM. One-way ANOVA with Bonferroni's Multiple Comparison Test was used to compare groups. ns = non-significant.

# Supporting Information References


Backs J, Backs T, Neef S, Kreusser MM, Lehmann LH, Patrick DM, Grueter CE, Qi X, Richardson JA, Hill JA, Katus HA, Bassel-Duby R, Maier LS, Olson EN (2009) The delta isoform of CaM kinase II is required for pathological cardiac hypertrophy and remodeling after pressure overload. *Proc Natl Acad Sci U S A* **106:** 2342-2347

Backs J, Song K, Bezprozvannaya S, Chang S, Olson EN (2006) CaM kinase II selectively signals to histone deacetylase 4 during cardiomyocyte hypertrophy. *J Clin Invest* **116:** 1853-1864

Goehringer C, Rutschow D, Bauer R, Schinkel S, Weichenhan D, Bekeredjian R, Straub V, Kleinschmidt JA, Katus HA, Muller OJ (2009) Prevention of cardiomyopathy in delta-sarcoglycan knockout mice after systemic transfer of targeted adeno-associated viral vectors. *Cardiovasc Res* **82:** 404-410

Kohlhaas M, Liu T, Knopp A, Zeller T, Ong MF, Bohm M, O'Rourke B, Maack C (2010) Elevated cytosolic Na+ increases mitochondrial formation of reactive oxygen species in failing cardiac myocytes. *Circulation* **121:** 1606-1613

Leuschner F, Dutta P, Gorbatov R, Novobrantseva TI, Donahoe JS, Courties G, Lee KM, Kim JI, Markmann JF, Marinelli B, Panizzi P, Lee WW, Iwamoto Y, Milstein S, Epstein-Barash H, Cantley W, Wong J, Cortez-Retamozo V, Newton A, Love K, Libby P, Pittet MJ, Swirski FK, Koteliansky V, Langer R, Weissleder R, Anderson DG, Nahrendorf M (2011) Therapeutic siRNA silencing in inflammatory monocytes in mice. *Nature biotechnology* **29:** 1005-1010

Mela L, Seitz S (1979) Isolation of mitochondria with emphasis on heart mitochondria from small amounts of tissue. *Methods in enzymology* **55:** 39-46

Pacher P, Nagayama T, Mukhopadhyay P, Batkai S, Kass DA (2008) Measurement of cardiac function using pressure-volume conductance catheter technique in mice and rats. *Nat Protoc* **3:** 1422-1434

Schinkel S, Bauer R, Bekeredjian R, Stucka R, Rutschow D, Lochmuller H, Kleinschmidt JA, Katus HA, Muller OJ (2012) Long-Term Preservation of Cardiac Structure and Function After Adeno-Associated Virus Serotype 9-Mediated Microdystrophin Gene Transfer in mdx Mice. *Human gene therapy* **23:** 566-575

Shioura KM, Geenen DL, Goldspink PH (2007) Assessment of cardiac function with the pressure-volume conductance system following myocardial infarction in mice. *Am J Physiol Heart Circ Physiol* **293:** H2870-2877

Wei AC, Aon MA, O'Rourke B, Winslow RL, Cortassa S (2011) Mitochondrial energetics, pH regulation, and ion dynamics: a computational-experimental approach. *Biophys J* **100:** 2894-2903
