# Supplementary figures and images for: CaM Kinase II mediates maladaptive post-infarct remodeling and pro-inflammatory chemoattractant signaling but not acute myocardial ischemia/reperfusion injury
Source: EMBO Mol Med. 2014 Sep 5;6(10):1231–45. doi: 10.15252/emmm.201403848 (PMC4287929; doi:10.15252/emmm.201403848)

Full uncut gels of Supporting Information Fig 4:

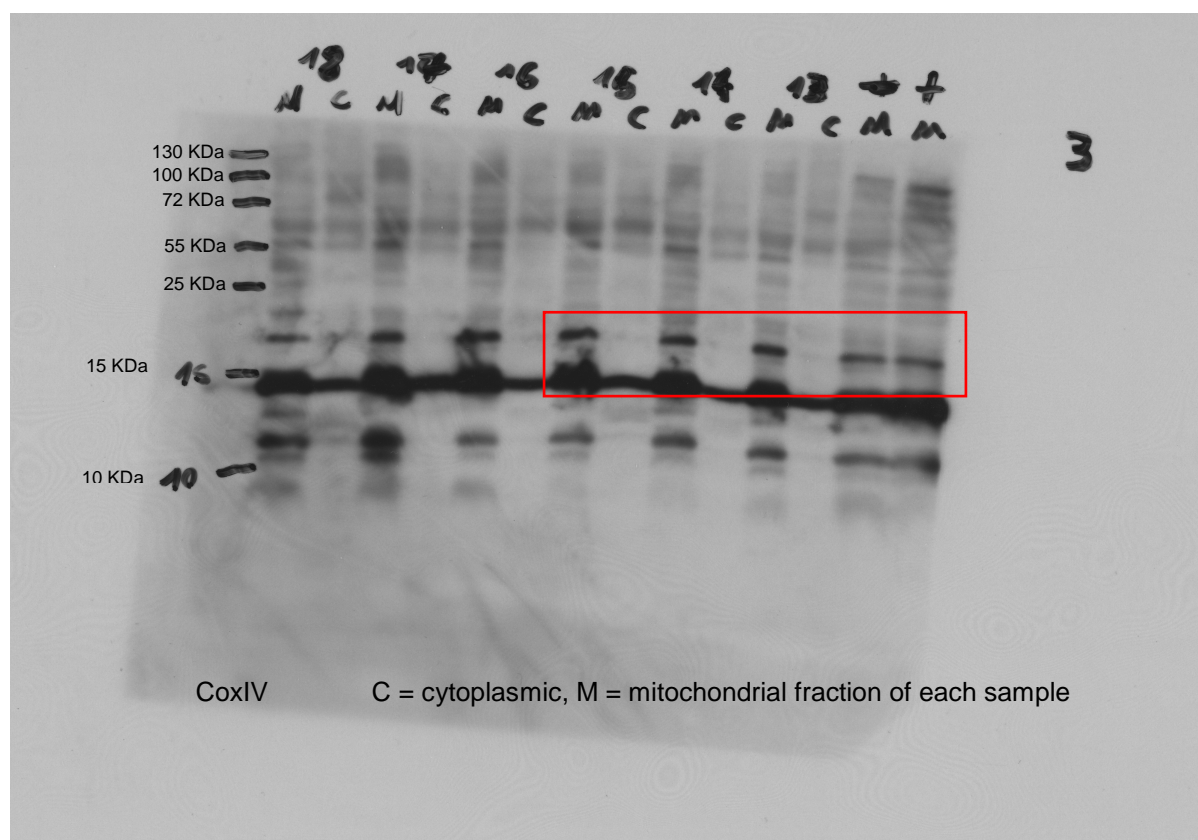

Supplement: Supplementary file 2 [file emmm0006-1231-sd2.pdf]

Full uncut gels of Supporting Information Figure 5A:

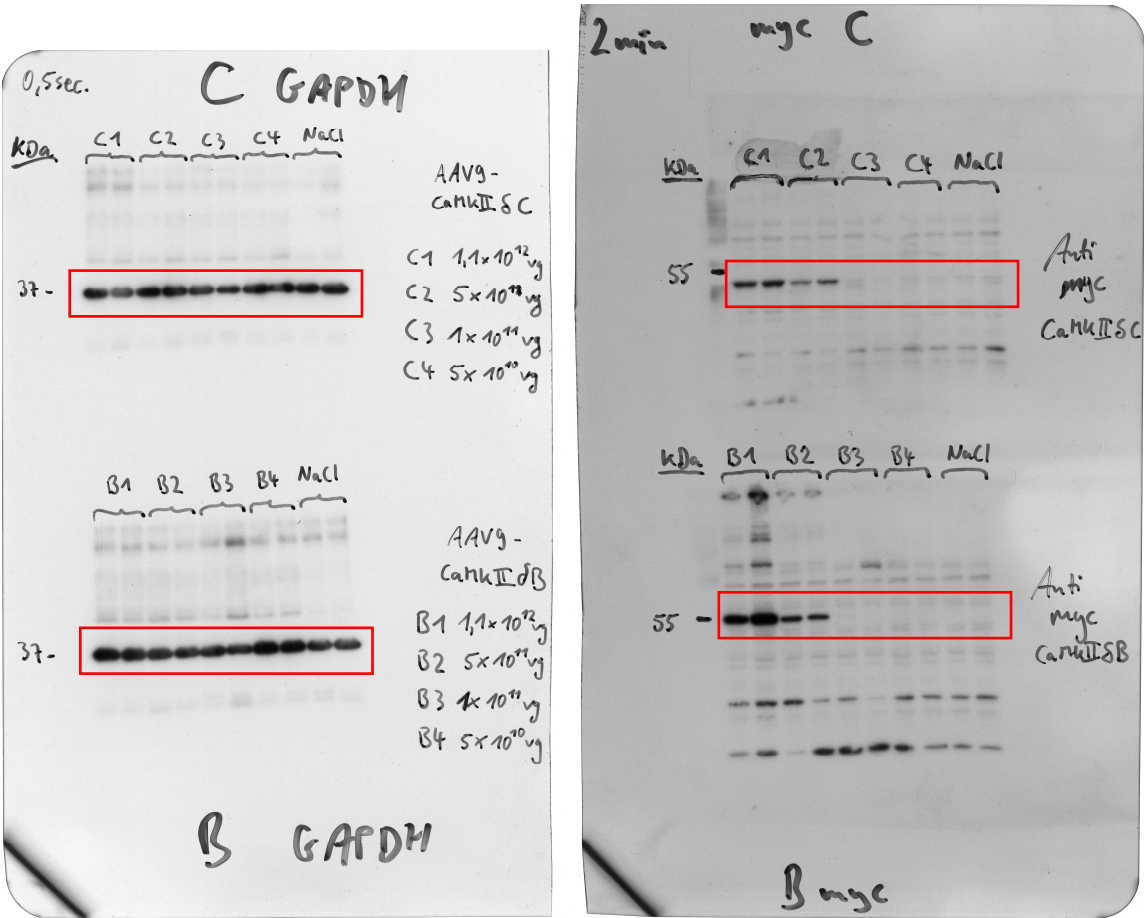

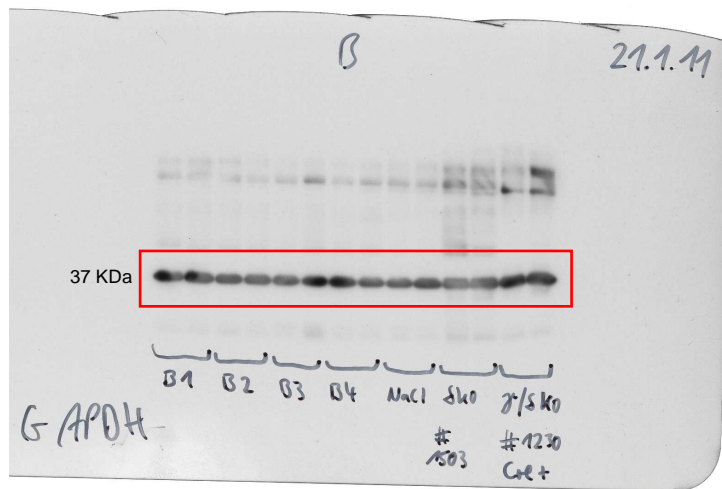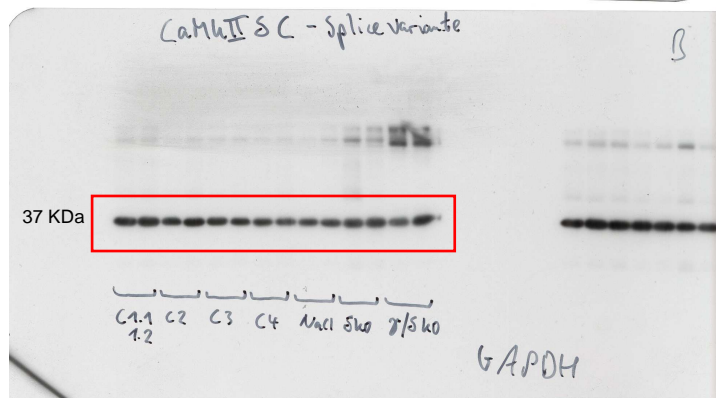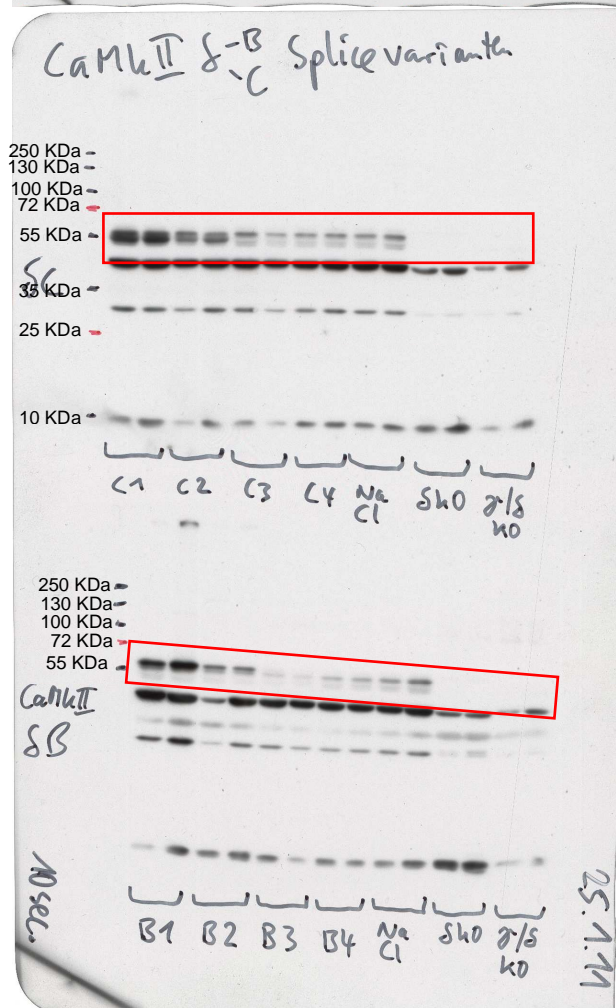

Supplement: Supplementary file 3 [file emmm0006-1231-sd3.pdf]

Full uncut gels of Supporting Information Figure 5C:

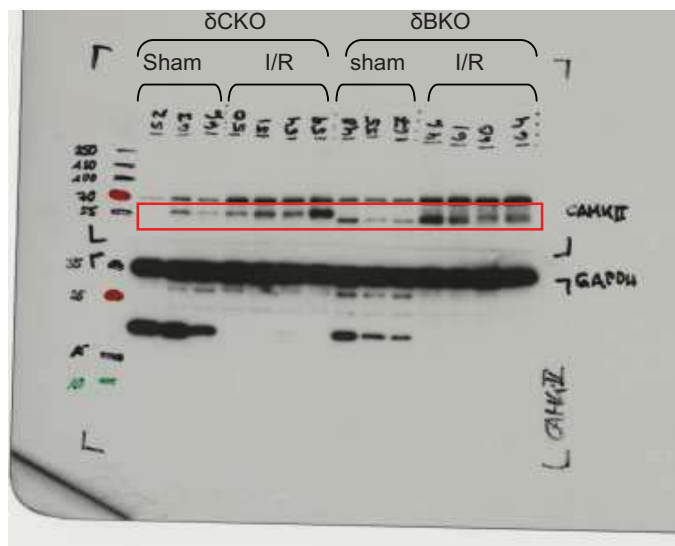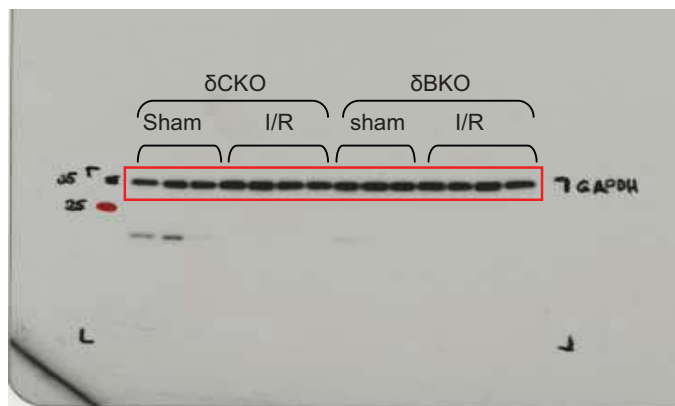

Supplement: Supplementary file 4 [file emmm0006-1231-sd4.pdf]

Full uncut gels of Supporting Information Fig 5D:

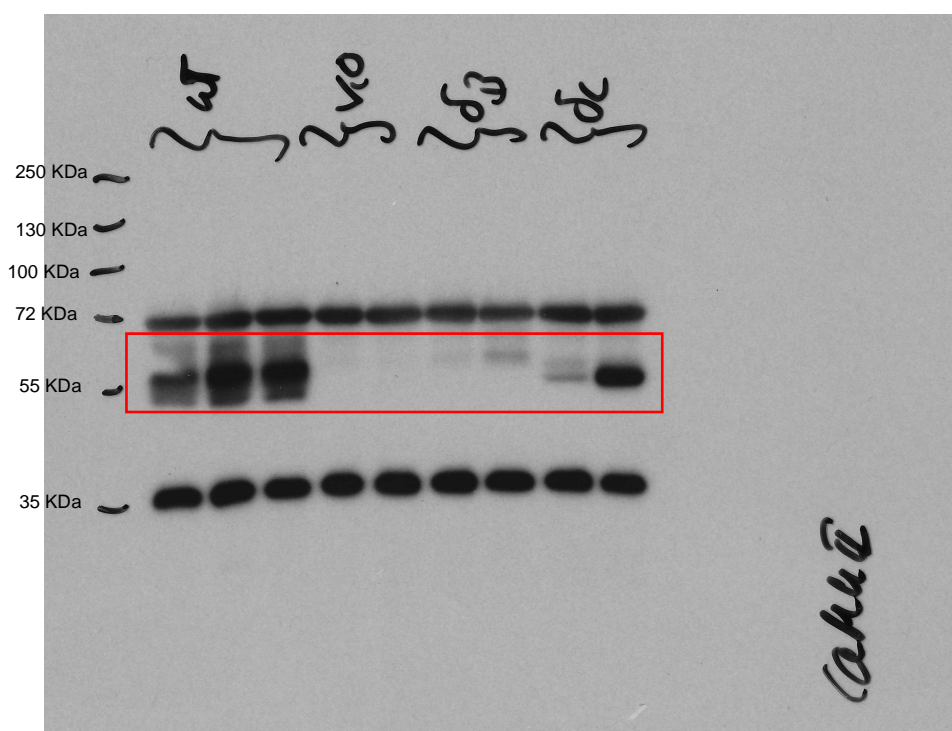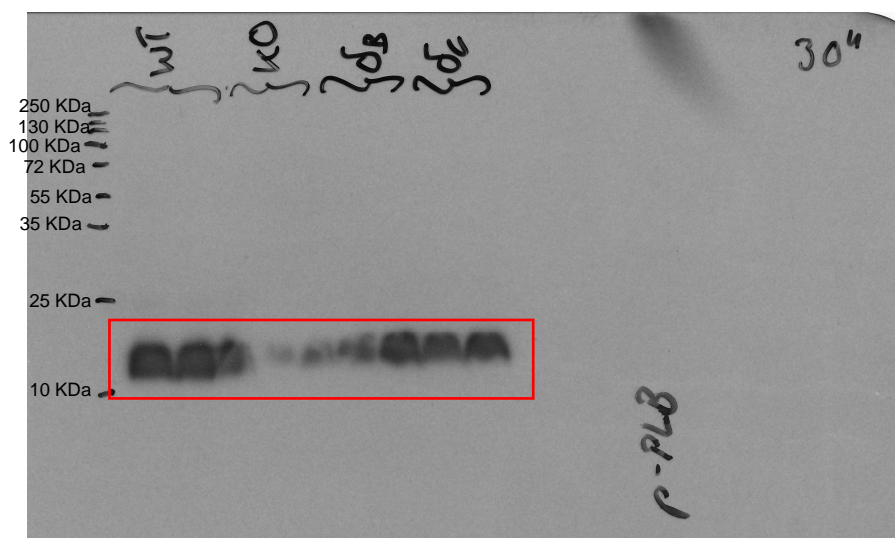

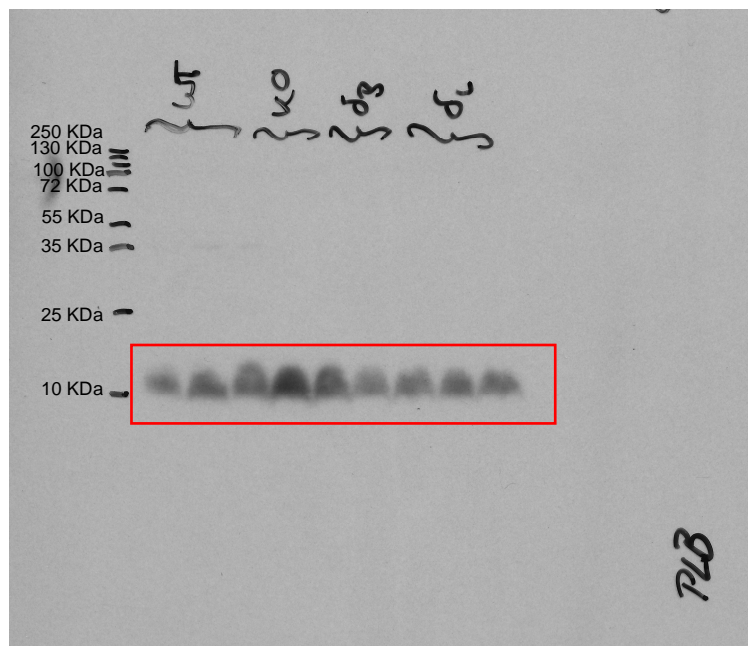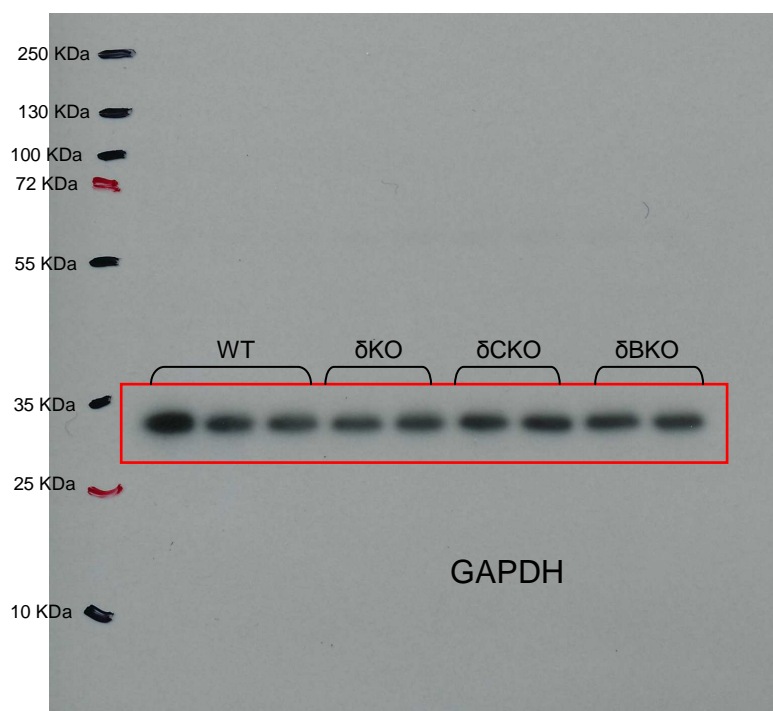

Supplement: Supplementary file 5 [file emmm0006-1231-sd5.pdf]

Full uncut gels of Supporting Information Fig 6:

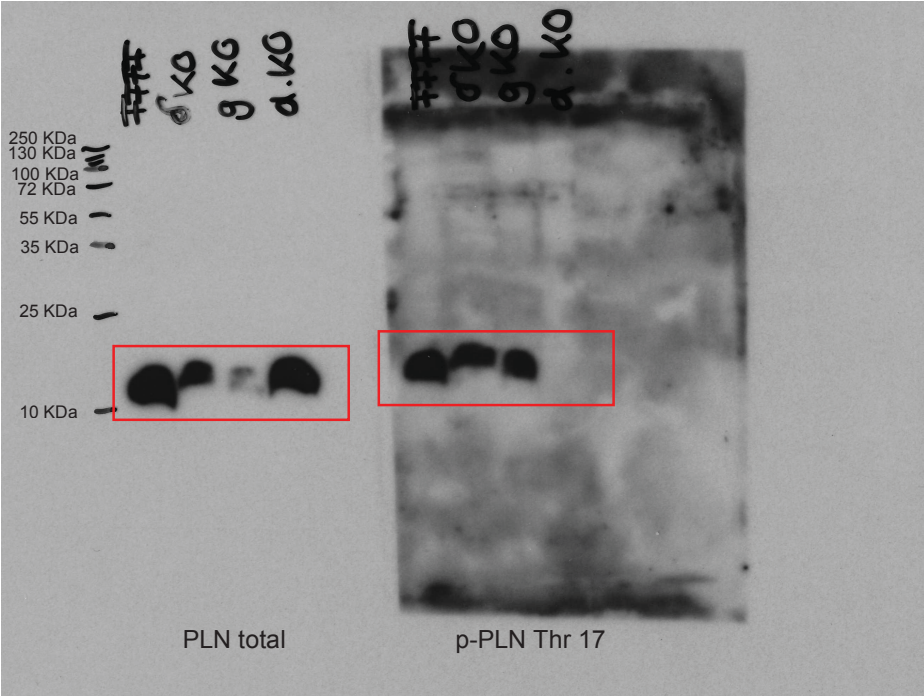

Supplement: Supplementary file 6 [file emmm0006-1231-sd6.pdf]

## Full uncut gels of Figure 2A:

### Cytosol

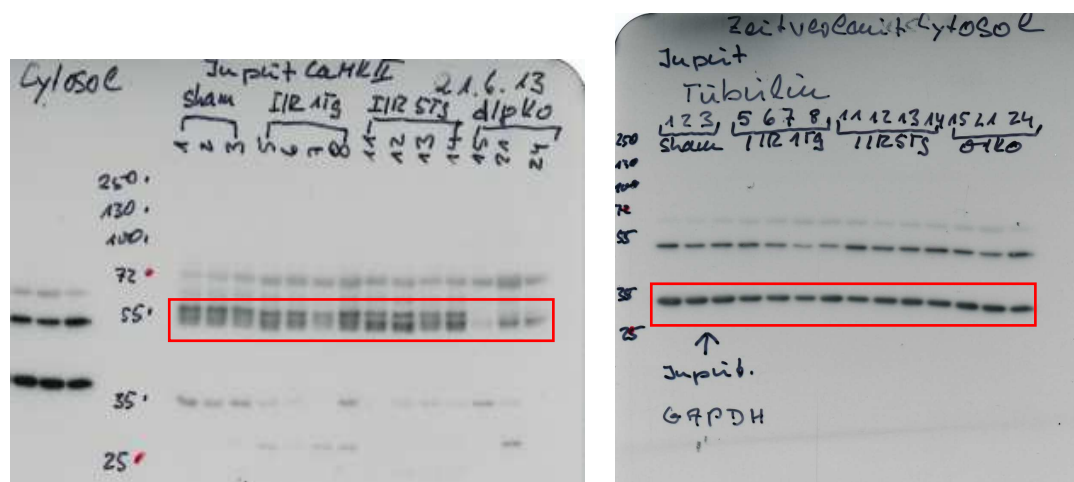

### Mitochondria

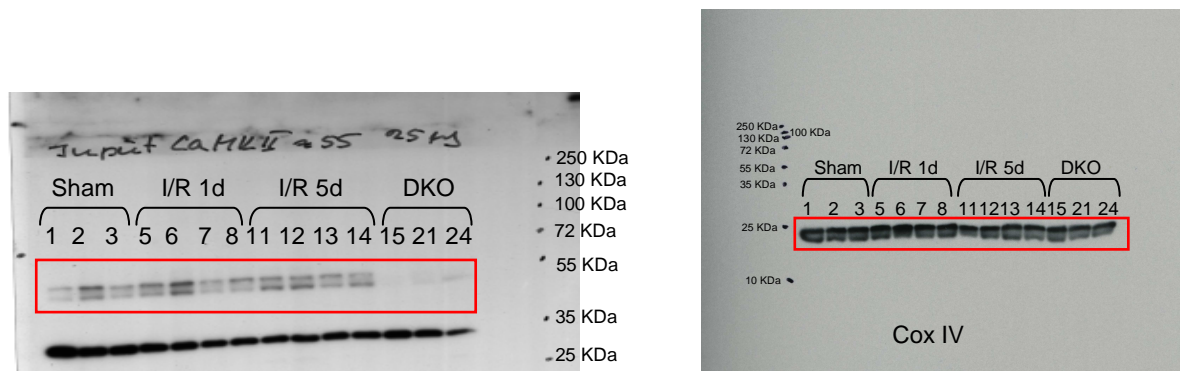

Supplement: Supplementary file 9 [file emmm0006-1231-sd9.pdf]

Full uncut gels of Figure 3:

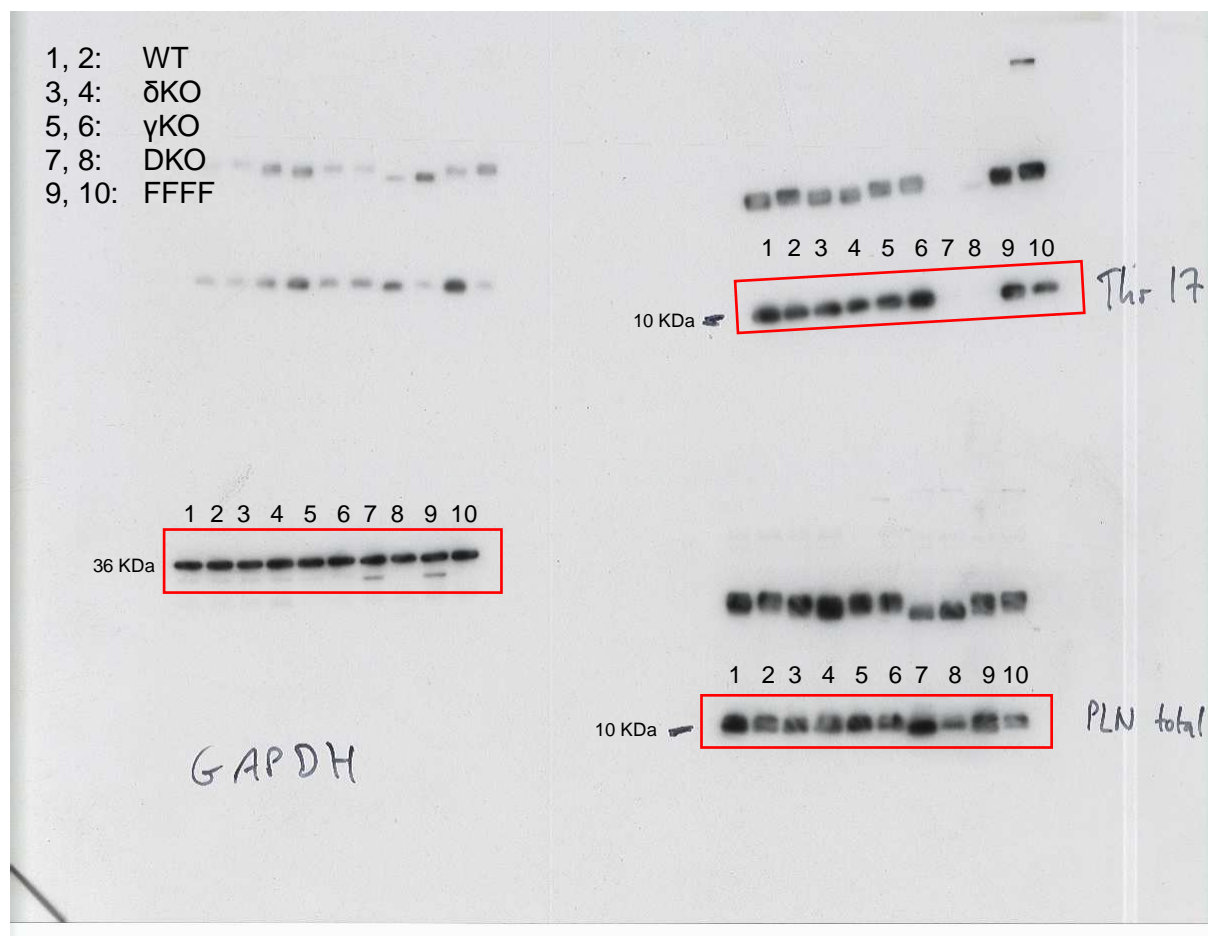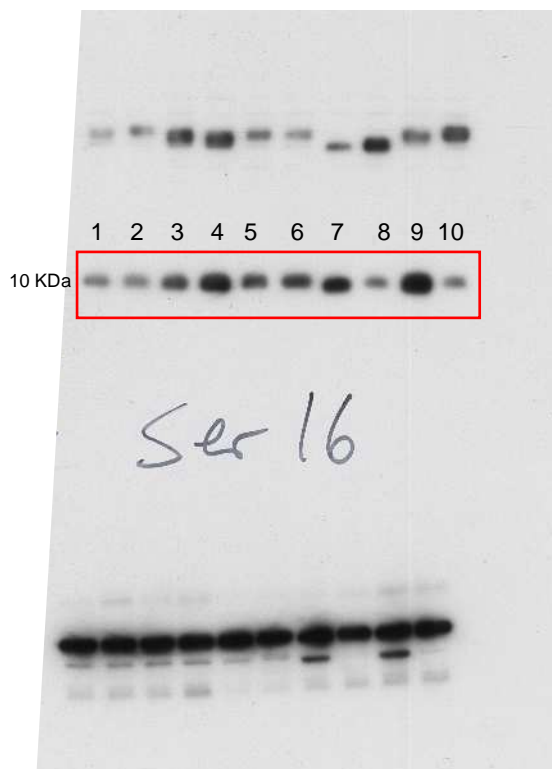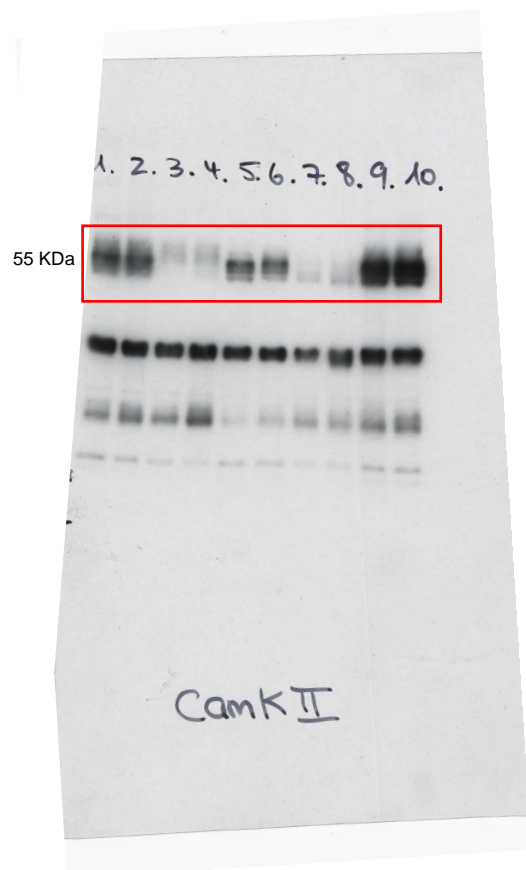

Supplement: Supplementary file 10 [file emmm0006-1231-sd10.pdf]
