# Supplementary material for: CaM Kinase II mediates maladaptive post-infarct remodeling and pro-inflammatory chemoattractant signaling but not acute myocardial ischemia/reperfusion injury
Source: EMBO Mol Med. 2014 Sep 5;6(10):1231–45. doi: 10.15252/emmm.201403848 (PMC4287929; doi:10.15252/emmm.201403848)
Supplement: Supplementary file 7 [file emmm0006-1231-sd7.pdf]

Full uncut gels of Supporting Information Fig 7B (12 weeks time point):

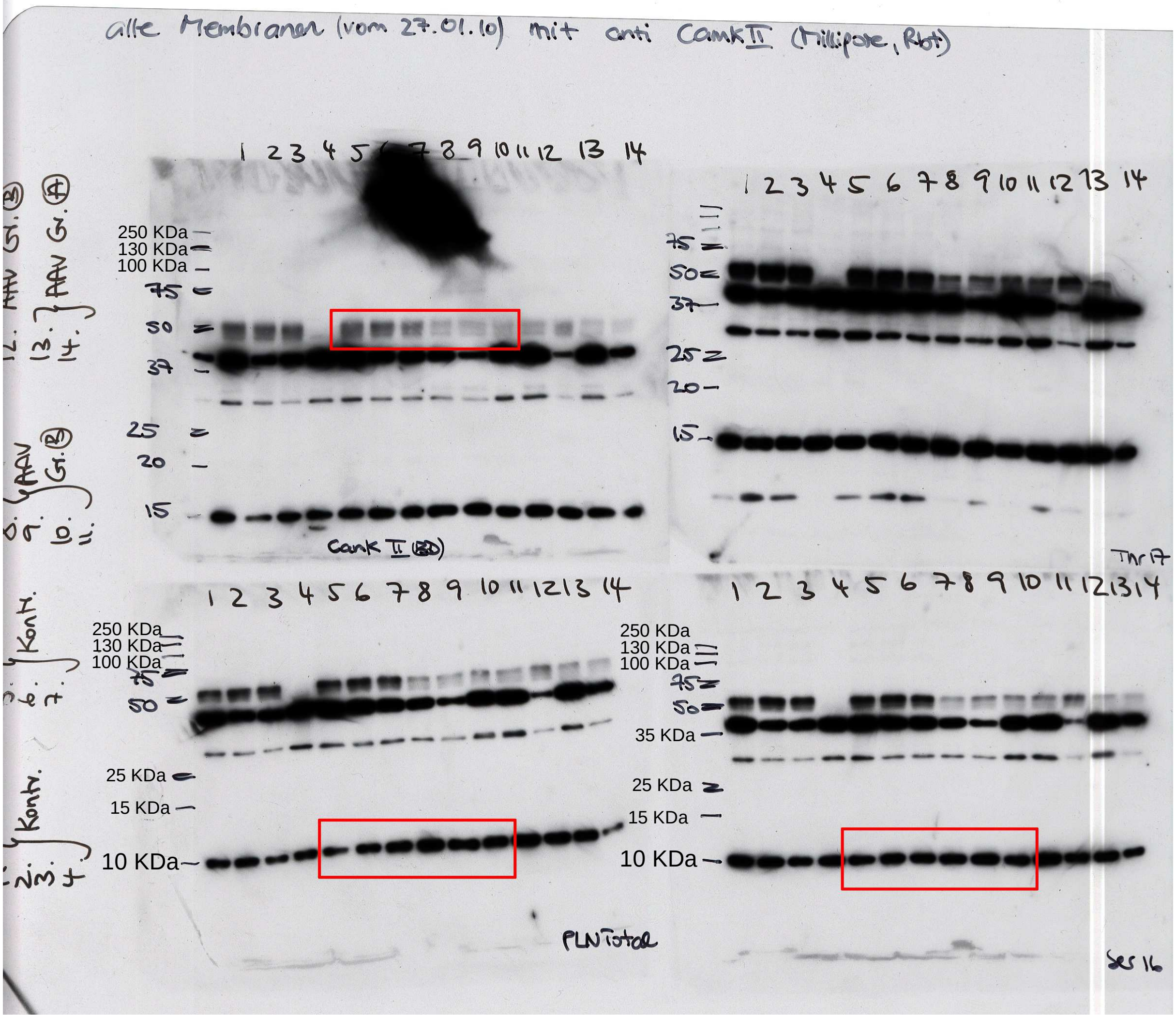

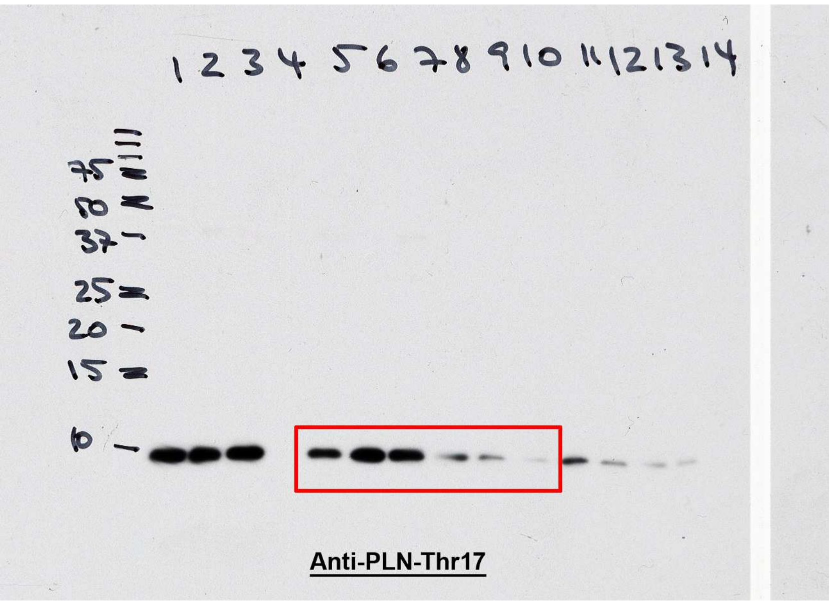

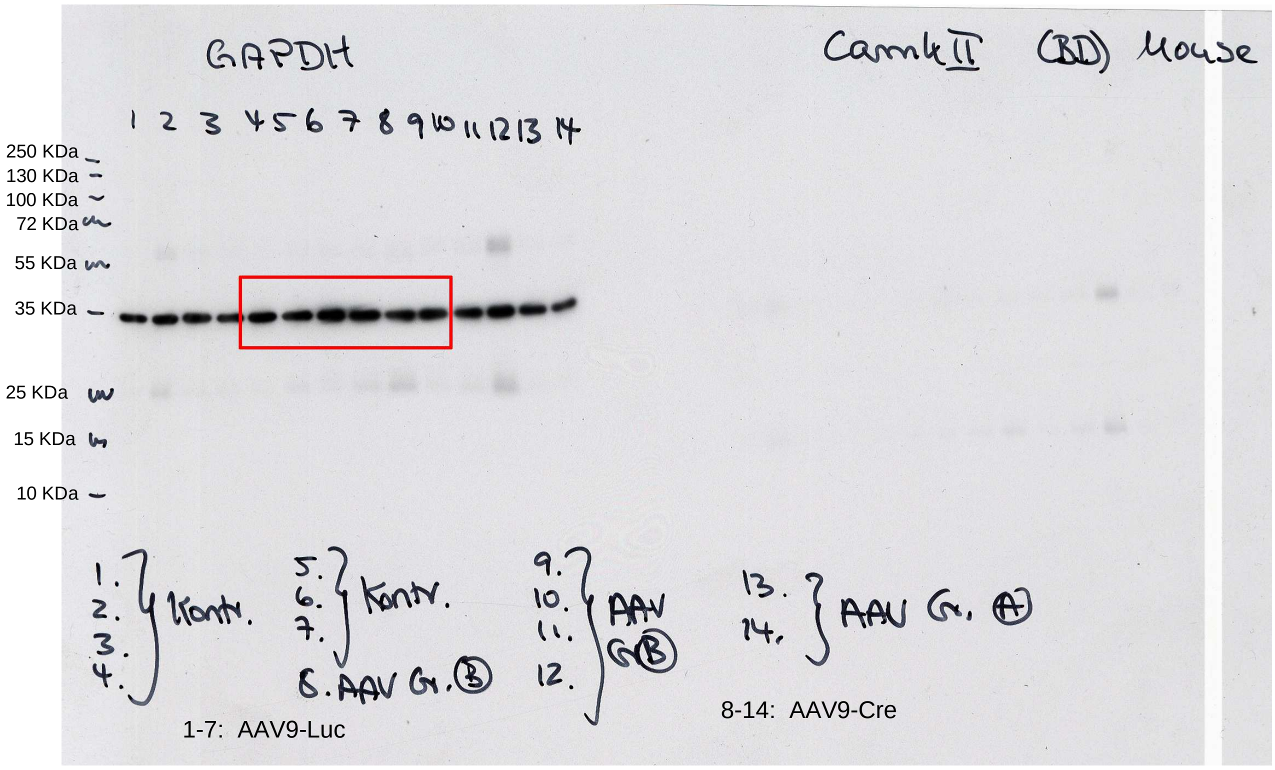
GAPDH
CamkII (BD) Mouse
1 2 3 4 5 6 7 8 9 10 11 12 13 14
250 KDa
130 KDa
100 KDa
72 KDa
55 KDa
35 KDa
25 KDa
15 KDa
10 KDa
1. 2. 3. 4. Kontr.
5. 6. 7. Kontr.
8. AAV Gr. B
9. 10. 11. 12. AAV Gr B
13. 14. AAV Gr. A
1-7: AAV9-Luc
8-14: AAV9-Cre
