# Supplementary material for: CaM Kinase II mediates maladaptive post-infarct remodeling and pro-inflammatory chemoattractant signaling but not acute myocardial ischemia/reperfusion injury
Source: EMBO Mol Med. 2014 Sep 5;6(10):1231–45. doi: 10.15252/emmm.201403848 (PMC4287929; doi:10.15252/emmm.201403848)
Supplement: Supplementary file 8 [file emmm0006-1231-sd8.pdf]

EMBO Molecular Medicine

CaM Kinase II mediates maladaptive post-infarct remodeling and pro-inflammatory chemoattractant signaling but not acute myocardial ischemia/reperfusion injury

Martin Weinreuter, Michael M.Kreusser, Jan Beckendorf, Friederike C. Schreiter, Florian Leuschner, Lorenz H. Lehmann, Kai P. Hofmann, Julia S. Rostosky, Nathalie Diemert, Chang Xu, Hans Christian Volz, Andreas Jungmann, Alexander Nickel, Carsten Sticht, Norbert Gretz, Christoph Maack, Michael D. Schneider, Hermann-Josef Gröne, Oliver Müller, Hugo A. Katus and Johannes Backs

Corresponding author: Johannes Backs, University of Heidelberg

Review timeline:

Submission date:	10 January 2014
Editorial Decision:	10 February 2014
Revision received:	15 June 2014
Editorial Decision:	13 July 2014
Revision received:	28 July 2014
Editorial Decision:	29 July 2014
Revision received:	30 July 2014
Accepted:	01 August 2014

Transaction Report:

(Note: With the exception of the correction of typographical or spelling errors that could be a source of ambiguity, letters and reports are not edited. The original formatting of letters and referee reports may not be reflected in this compilation.)

1st Editorial Decision 10 February 2014

Thank you for the submission of your manuscript to EMBO Molecular Medicine. We have now heard back from the three Reviewers whom we asked to evaluate your manuscript.
You will see that significant issues are raised that prevent us from considering publication at this time. I will not dwell into much detail, as the evaluations are detailed and self-explanatory and will just mention a few main points.

Although Reviewer 1 is more supportive of your work, s/he and Reviewer 2 point out that the study falls short on demonstrating the functions of CaMKII at later stages of cardiac remodelling. The two Reviewers suggest approaches to address this problem. We agree that this issue is a critical one and that without developing this aspect, the manuscript would lack the sufficient conceptual advance we would like to see in EMBO Molecular Medicine and would be limited to the negative data, which would be of rather specialistic interest. I should point out that Reviewer 2 also lists other very important experimental shortcomings and requests for clarification that require your action.

Reviewer 3 lists a number of potential experimental flaws and mentions the lack of sufficient experimental details to explain the potential discrepancy with respect to published evidence on the effect of CaMKIIdelta depletion on apoptosis in a similar model. These issues need to be addressed.

Considered all the above, while publication of the paper cannot be considered at this stage, we would be prepared to consider a substantially revised submission, with the understanding that the

Reviewers' concerns must be fully addressed with additional experimental data where appropriate and that acceptance of the manuscript will entail a second round of review.

Since the required revision in this case appears to require a significant amount of time, additional work and experimentation and might be technically challenging, I would understand if you chose to rather seek publication elsewhere at this stage. Should you do so, we would welcome a message to this effect.

***** Reviewer's comments *****

Referee #1 (Comments on Novelty/Model System):

The study by Weinreuter et al. used different mouse models to address the functions of the CaMKII isoforms under ischemia/reperfusion (I/R) condition. An extreme care was taken for each study design by performing a number of control experiments to derived at the conclusion that the isoforms of CaMKII do not play a major role immediately after I/R injury. However, the authors identified that cardiac function was better in CamKII DKO mice compared to FFFF control mice 5 weeks after I/R surgery to suggest that CaMKII is important during the process of post-infarct remodeling.

Up until the last section of results, the authors demonstrated very careful experiments. It must not be very easy to prove that there is no change in CamKII KO mice under I/R as negative results are always hard to prove than drastic changes that one could observe when KO mice were compared to control ones. Thus, this is really nice study being performed here.

The weak point of this manuscript is that the reduced infiltration of CD45+ leukocytes five days after I/R injury in DKO mice (Figure 6) was suspected to be the reason for reduced scar formation. In the Discussion section, the authors listed possible mechanisms for such biological phenomenon. As the authors concluded this section: "Thus, we speculate that CaMKII affects the communication between cardiomyocytes and leukocytes...2013). It will be of particular interest to identify target genes of these transcription factors that are secreted and may regulate leukocyte infiltration." However, no experimental evidence from the authors' side is provided. Would it not be possible to check for certain marker genes/proteins by isolating cardiomyocytes, fibroblasts, endothelial cells, and leukocytes by performing RT-PCR/Western blotting? Even, performing a global profiling by microarrays/RNA-seq would help to add more mechanistic aspect to this currently well-done manuscript.

Referee #1 (Remarks):

The study by Weinreuter et al. used different mouse models to address the functions of the CaMKII isoforms under ischemia/reperfusion (I/R) condition. An extreme care was taken for each study design by performing a number of control experiments to derived at the conclusion that the isoforms of CaMKII do not play a major role immediately after I/R injury. However, the authors identified that cardiac function was better in CamKII DKO mice compared to FFFF control mice 5 weeks after I/R surgery to suggest that CaMKII is important during the process of post-infarct remodeling.

Up until the last section of results, the authors demonstrated very careful experiments. It must not be very easy to prove that there is no change in CamKII KO mice under I/R as negative results are always hard to prove than drastic changes that one could observe when KO mice were compared to control ones. Thus, this is really nice study being performed here.

The weak point of this manuscript is that the reduced infiltration of CD45+ leukocytes five days after I/R injury in DKO mice (Figure 6) was suspected to be the reason for reduced scar formation. In the Discussion section, the authors listed possible mechanisms for such biological phenomenon. As the authors concluded this section: "Thus, we speculate that CaMKII affects the communication between cardiomyocytes and leukocytes...2013). It will be of particular interest to identify target genes of these transcription factors that are secreted and may regulate leukocyte infiltration." However, no experimental evidence from the authors' side is provided. Would it not be possible to check for certain marker genes/proteins by isolating cardiomyocytes, fibroblasts, endothelial cells, and leukocytes by performing RT-PCR/Western blotting? Even, performing a global profiling by

microarrays/RNA-seq would help to add more mechanistic aspect to this currently well-done manuscript.

Referee #2 (Remarks):

The manuscript by Weinreuter investigates CaMKII isoforms/splice variants during different phase of cardiac remodeling in an I/R MI model. This is a well designed and executed study focusing mostly on early phase with less emphasis invested into the later phases. The study is of importance because it discussed a controversial topic related to the role of CaMKII during the different phases of cardiac remodeling. In addition the authors identified an important role of CaMKII in the later phase. The study is restricted to mouse work and its implication in a clinical setting is not clearly demonstrated.
Overall there are several issues that need to be addressed.
Figure 1/2: It is unclear why the authors use a global ko mouse model when later on they are using a CM-specific model which should be the preferential model.
The viral overexpression experiments for B/C forms are problematic. Although similar expression levels are achieved it is unclear what is happening on a cellular level, which determines the validation of their system compare to the in vivo situation.
The viral Cre overexpression model is problematic as well. It is unclear why the ko only becomes obvious after 12 week? This may indicate of target effects of their gene delivery model. Genetic inducible Cre systems might be an alternative.
The study falls short on the second part investigating the functions of CaMKII at later stages of cardiac remodeling. This needs to be rigorously investigated with focus on inflammation, scare formation / fibrotic response and cardiomyocyte adaption at later phases.

Referee #3 (Remarks):

This manuscript investigates the role of CaMKII specific isoforms as a mediator of ischemic myocardial injury and adverse cardiac remodeling in murine models, and conclude that CaMKII regulate the process of post-infarct remodeling, rather than acute I/R injury.

General considerations
The topic of the manuscript is very specific, and would better fit a journal with a more specific focus.
The quality of Tunel assays is in general very poor throughout the manuscript, raising doubts about the conclusions.
The western blots in supplemental material need improvement, especially referring to CaMKII delta B KO.
Results

CaMKIIδ does not regulate myocardial damage shortly after I/R injury.
The conclusion of these results is based upon the TUNEL assays in Fig. 1. According to the authors, the results support that genetic deletion of CaMKII delta does not exert any effect on apoptosis following ischemia-reperfusion damage. It has been extensively and convincingly demonstrated (Brown, 2008; Ling et al, 2013) that genetic deletion of CaMKII delta isoform reduces apoptosis in the same model: the authors do not provide sufficient insights to explain the discrepancies, also showing technical weaknesses into the Tunel assays.

Role of CaMKIIδ Splice Variants in Acute Myocardial I/R Injury.
Fig. 2C and F. The authors need to show controls of void adeno-associated vectors. Furthermore, delta-C CaMKII overexpression has been previously demonstrated to be associated with dilated myocardiopathy (Sossalla, 2011), whereas overexpression of delta-B CaMKII has been demonstrated to associate with increased apoptosis. In both cases, the authors do not address the discrepancy of their results.

Combined cardiomyocyte-specific deletion of CaMKIIδ/γ does not affect I/R injury.

The authors show results (Fig. 3) supporting that PLB phosphorylation is reduced only when they double knock delta and gamma CaMKII isoforms, whereas no reduction whatsoever is achieved by CaMKII delta, the main isoform expressed in their model. The authors should provide data on isolated cardiomyocite rather than on whole ventricular extract, and should measure CaMKII activity by alternative techniques (for example kinase assays).

Fig. 4, Fig. 5 and Fig. 6 all lack the wild type controls: no conclusions can be convincingly drawn by these results.

1st Revision - authors' response 15 June 2014

We would like to thank all the reviewers for their excellent comments that helped us to improve the quality of the manuscript. We performed new experiments to address the concerns. Most importantly, we applied a systematic approach to identify pro-inflammatory signalling pathways that may regulate leukocyte infiltration. Doing so, we identified CCL3 as a previously not identified CaMKII-dependent chemoattractant signaling molecule. We also performed new experiments to show that our CaMKII splicing variant re-expression approach was indeed functional and we also show in isolated cardiac myocytes that the double knockout of CaMKII delta and gamma results in complete prevention of PLB Thr17 phosphorylation. All changes in the revised manuscript are highlighted in green.

Response to Referee #1

The weak point of this manuscript is that the reduced infiltration of CD45+ leukocytes five days after I/R injury in DKO mice (Figure 6) was suspected to be the reason for reduced scar formation. In the Discussion section, the authors listed possible mechanisms for such biological phenomenon. As the authors concluded this section: "Thus, we speculate that CaMKII affects the communication between cardiomyocytes and leukocytes...2013). It will be of particular interest to identify target genes of these transcription factors that are secreted and may regulate leukocyte infiltration." However, no experimental evidence from the authors' side is provided. Would it not be possible to check for certain marker genes/proteins by isolating cardiomyocytes, fibroblasts, endothelial cells, and leukocytes by performing RT-PCR/Western blotting? Even, performing a global profiling by microarrays/RNA-seq would help to add more mechanistic aspect to this currently well-done manuscript.

We thank the reviewer for this comment and we fully agree that it is of particular interest to identify target genes involved in leukocyte infiltration in a CaMKII-dependent manner. We followed the reviewer's suggestion and performed an unbiased RNA microarray analysis in samples from I/R-operated mouse hearts at day 1 and day 5 after I/R injury as compared to sham-operated hearts. We found inflammatory chemokine pathways to be attenuated in DKO as compared to FFFF. The systems biological approach that we followed is now described in Suppl. Fig. 9, Figure 6 and Suppl. Fig. 10. Because the microarray analysis was done in whole heart samples, we tested the top regulated genes of the "Chemokine Signaling Pathway" in cultured cardiomyocytes from CaMKII KO mice or in wild type cardiomyocytes after adenoviral expression with active CaMKII (or after hypoxia (Figure 6). We could show that the pro-inflammatory chemoattractant ligand CCL3 but not the components of inflammatory cells were induced by CaMKII in cardiomyocytes, indicating a cardiomyocyte autonomous effect with regard to CCL3. Moreover, we provide evidence that hypoxia alone induces CCL3 gene expression and its protein expression in the supernatant of cardiomyocytes (Figure 6). Taken together, we demonstrate reduced leukocyte infiltration five days after I/R in DKO mice, which was preceded by a reduced expression of the chemoattractant molecule CCL3. This approach is now described in detail in the result part:

"To identify CaMKII-dependent inflammatory pathways, we performed unbiased and systematic RNA analyses by Affymetrix chip arrays in cardiac samples at 1 day and 5 days after I/R injury versus sham-operated hearts. Gene Set Enrichment Analysis (GSEA) was then used to determine whether defined sets of genes exhibit a statistically significant bias in their distribution within a ranked gene list (Subramanian et al, 2005). Pathways belonging to specific cell functions were

obtained from public external databases (KEGG PATHWAY Database, http://www.genome.jp/kegg/) to perform pathway analyses. Doing so, we identified several inflammatory pathways to be attenuated in DKO as compared to FFFF (Suppl. Figure 9A). Intriguingly, the "Chemokine Signaling Pathway" was attenuated at both 1 and 5 days after I/R. Whereas several ligands to inflammatory cells were attenuated 1 and 5 day after I/R, the expression of intracellular components of inflammatory cells were mostly attenuated 5 days after I/R, confirming an attenuated infiltration of inflammatory cells at this time point (Suppl. Figure 9B). We then validated the top 5 attenuated genes of the "Chemokine Signaling Pathway" at the 1 and 5 day time point after I/R by RT-PCR (Suppl. Figure 9C) and we could confirm an attenuated gene expression of CCL3, CCR1, CXCR2, PPBP, HCK, FGR and CCL6. However, CXCL3 could not be detected by RT-PCR and VAV1 and CCL2 could not formally be validated, although a not statistically significant trend towards attenuated expression could be noted. Based on the observation that 5 days after I/R more intracellular components of inflammatory cells were enriched, we speculated that these changes are rather indirectly and secondarily mediated by CaMKII by non-cardiomyocyte autonomous effects. Vice versa, we envisioned that the chemoattractant ligands, which were already attenuated 1 day after I/R, might be induced by CaMKII in cardiomyocytes. Thus, we tested whether adenoviral expression of active CaMKII (CaMKII-T287D) was sufficient to induce mRNA expression of selected genes of the "Chemokine Signaling Pathway". Strikingly, CaMKII induced only expression of members of the chemokine (C-C motif) ligand family (Figure 6C), in particular CCL2 (also known as monocyte chemotactic protein 1, MCP1) and even more pronounced CCL3 (also known as macrophage inflammatory protein 1 alpha, MIP-1 alpha) but not CCR1, VAV1, HCK or FGR (Suppl. Figure 10), which according to the KEGG PATHWAY Database are all intracellular components of inflammatory cells, supporting our hypothesis that these genes are rather indirectly and secondarily regulated by CaMKII via non-cardiomyocyte autonomous effects. To rule out an artificial effect due to adenovial infections of NRVMs we used another adenovirus with a high titer (10 MOI), in this case NFAT-GFP, as a control and found no effect on CCL2 and CCL3 expression. In these experiments, CaMKII expression was confirmed by RT-PCR (3 MOI led to a 18-fold increase in CaMKII expression and 10 MOI to a 114-fold increase).
To further support these data, we asked whether CaMKII is also required for CCL2 and CCL3 gene expression in isolated cardiomyocytes. Thus, we isolated adult mouse ventricular myocytes (AMVMs) from FFFF and DKO and applied hypoxia followed by 8 hours normoxia (Figure 6D). We found that CCL3 but not CCL2 was significantly up-regulated by hypoxia, which was clearly attenuated in DKO cardiomyocytes. Taken together, CaMKII is sufficient and required for cardiomyocyte autonomous CCL3 expression. To test whether an increased gene expression of CCL2 and CCL3 results in an increased secretion of the protein, we performed hypoxia experiments in NRVMs (Figure 6E + F) and found that an increase in CCL2 and CCL3 mRNA preceded an actual increase of CCL2 and CCL3 protein in the supernatant of NRVMs 24 hours after hypoxia, potentially explaining the late effects on infiltration of CD45+ cells."

Response to Referee #2

Figure 1/2: It is unclear why the authors use a global ko mouse model when later on they are using a CM-specific model which should be the preferential model.

We appreciate the reviewer's comments. In the first experiments, we investigated the role of CaMKII delta – the isoform with highest expression level in cardiomyocytes – in I/R injury, because we hypothesized that a CaMKII delta KO leads to smaller infarct sizes. We started with global KO mice because we hypothesized that the global deletion provides the highest chance to obtain significant differences in case CaMKII delta would regulate acute I/R injury. However, surprisingly we obtained negative data in the single KO models (for CaMKII delta and gamma, respectively). Thus, we aimed to investigate a double knock-out for both isoforms, delta and gamma, to rule out redundant effects in the single KO models. However, the combined global deletion of CaMKII delta/gamma resulted in postnatal lethality. These findings are unpublished and the reason of lethality is currently under investigation (first evidence suggests that the lethality bases on CaMKII deletion in neural crest cells). Thus, we needed to use a cardiomyocyte-specific model to investigate potential redundant roles of CaMKII delta and gamma. This is stated on page 8:

"Because the combined global deletion of CaMKIIδ and γ resulted in early postnatal lethality, we generated double floxed CaMKIIδ/γ mice (CaMKIIγ$^{loxP/loxP}$; CaMKIIδ$^{loxP/loxP}$, here termed as FFFF) and crossed them with mice expressing Cre-recombinase under the control of the cardiomyocyte-specific α-MHC-promoter (Agah et al, 1997) to receive cardiomyocyte-specific CaMKIIδ/γ double KO mice (DKO)."

The viral overexpression experiments for B/C forms are problematic. Although similar expression levels are achieved it is unclear what is happening on a cellular level, which determines the validation of their system compare to the in vivo situation.

We agree that it is important to show the re-expressed slicing variants are functional. Therefore, we performed additional Western Blot analyses to test whether the deltaB and deltaC splice variants affect Phospholamban phosphorylation at the typical CaMKII phosphosite Threonin 17. This site was found to be hypophosphorylated in KO mice. But indeed both AAV-mediated CaMKII deltaB and in CaMKII deltaC expression increased phospholamban Thr-17 phosphorylation. These results are now shown and quantified in Suppl Fig 5D. This result is also shown here:

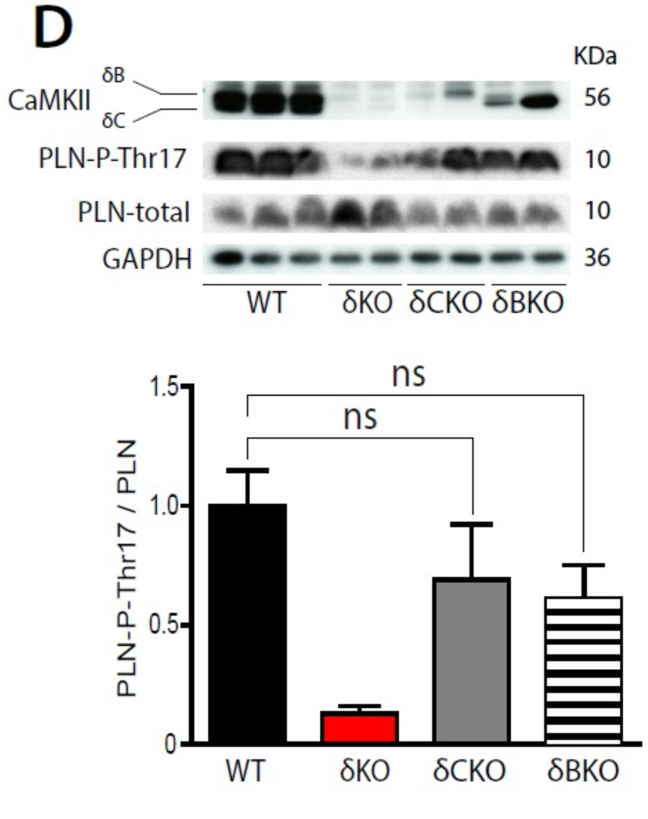

The viral Cre overexpression model is problematic as well. It is unclear why the ko only becomes obvious after 12 week? This may indicate of target effects of their gene delivery model. Genetic inducible Cre systems might be an alternative.

We chose the AAV9 Cre inducible KO system to avoid Cre-associated transient cardiomyopathy as it was reported for an alternative approach, in which tamoxifen is used in combination with Mer-Cre-Mer transgenic mice (Koitabashi et al, 2009; Molkentin & Robbins, 2009). The model described here is a straight-forward approach to circumvent unintended side-effects associated with e.g. the tamoxifen inducible KO model. This could be shown by the group of Oliver Müller (paper currently under review). To specifically answer the question of the reviewer, we further investigated the time course of efficient Cre expression after i.v. tail vein injection. We conducted time course experiments in ROSA26-LacZ mice, which demonstrate that efficient Cre-mediated LacZ reporter gene expression can only be observed from week 5 on, with its maximum around week 10 (Figure

removed as [by the Authors]. These results support our findings in the AAV9 Cre CaMKII double KO system (iDKO), which only shows efficient gene take-out of CaMKII at 12 weeks after i.v. injection (Suppl. Fig 6B). We suspect that this "relative slow time course" of recombination is due to a more moderate expression as compared to e.g. the Mer-Cre-Mer system. This "relative slow time course" accounts most likely also for the missing cardiotoxic effects (paper currently under review).

The study falls short on the second part investigating the functions of CaMKII at later stages of cardiac remodeling. This needs to be rigorously investigated with focus on inflammation, scare formation / fibrotic response and cardiomyocyte adaption at later phases.

We completely agree and we also thank reviewer 2 for this advice. As already mentioned in the response to reviewer 1, we performed extensive new experimentation to focus on the functions of CaMKII on inflammation: We performed an unbiased RNA microarray analysis in samples from I/R-operated mouse hearts at day 1 and day 5 after I/R injury as compared to sham-operated hearts. We found inflammatory chemokine pathways to be attenuated in DKO as compared to FFFF. The systems biological approach that we followed is now described in Suppl. Fig. 9, Figure 6 and Suppl. Fig. 10. Because the microarray analysis was done in whole heart samples, we tested the top regulated genes of the "Chemokine Signaling Pathway" in cultured cardiomyocytes from CaMKII KO mice or in wild type cardiomyocytes after adenoviral expression with active CaMKII (or after hypoxia (Figure 6). We could show that the pro-inflammatory chemoattractant ligand CCL3 but not the components of inflammatory cells were induced by CaMKII in cardiomyocytes, indicating a cardiomyocyte autonomous effect with regard to CCL3. Moreover, we provide evidence that hypoxia alone induces CCL3 gene expression and its protein expression in the supernatant of cardiomyocytes (Figure 6). Taken together, we demonstrate reduced leukocyte infiltration five days after I/R in DKO mice, which was preceded by a reduced expression of the chemoattractant molecule CCL3. This approach is now described in detail in the result part:

"To identify CaMKII-dependent inflammatory pathways, we performed unbiased and systematic RNA analyses by Affymetrix chip arrays in cardiac samples at 1 day and 5 days after I/R injury versus sham-operated hearts. Gene Set Enrichment Analysis (GSEA) was then used to determine whether defined sets of genes exhibit a statistically significant bias in their distribution within a ranked gene list (Subramanian et al, 2005). Pathways belonging to specific cell functions were obtained from public external databases (KEGG PATHWAY Database, http://www.genome.jp/kegg/) to perform pathway analyses. Doing so, we identified several inflammatory pathways to be attenuated in DKO as compared to FFFF (Suppl. Figure 9A). Intriguingly, the "Chemokine Signaling Pathway" was attenuated at both 1 and 5 days after I/R. Whereas several ligands to inflammatory cells were attenuated 1 and 5 day after I/R, the expression of intracellular components of inflammatory cells were mostly attenuated 5 days after I/R, confirming an attenuated infiltration of inflammatory cells at this time point (Suppl. Figure 9B). We then validated the top 5 attenuated genes of the "Chemokine Signaling Pathway" at the 1 and 5 day time point after I/R by RT-PCR (Suppl. Figure 9C) and we could confirm an attenuated gene expression of CCL3, CCR1, CXCR2, PPBP, HCK, FGR and CCL6. However, CXCL3 could not be detected by RT-PCR and VAV1 and CCL2 could not formally be validated, although a not statistically significant trend towards attenuated expression could be noted. Based on the observation that 5 days after I/R more intracellular components of inflammatory cells were enriched, we speculated that these changes are rather indirectly and secondarily mediated by CaMKII by non-cardiomyocyte autonomous effects. Vice versa, we envisioned that the chemoattractant ligands, which were already attenuated 1 day after I/R, might be induced by CaMKII in cardiomyocytes. Thus, we tested whether adenoviral expression of active CaMKII (CaMKII-T287D) was sufficient to induce mRNA expression of selected genes of the "Chemokine Signaling Pathway". Strikingly, CaMKII induced only expression of members of the chemokine (C-C motif) ligand family (Figure 6C), in particular CCL2 (also known as monocyte chemotactic protein 1, MCP1) and even more pronounced CCL3 (also known as macrophage inflammatory protein 1 alpha, MIP-1 alpha) but not CCR1, VAV1, HCK or FGR (Suppl. Figure 10), which according to the KEGG PATHWAY Database are all intracellular components of inflammatory cells, supporting our hypothesis that these genes are rather indirectly and secondarily regulated by CaMKII via non-cardiomyocyte autonomous effects. To rule out an artificial effect due to adenovial infections of NRVMs we used another adenovirus with a high titer (10 MOI), in this case NFAT-GFP, as a control and found no

effect on CCL2 and CCL3 expression. In these experiments, CaMKII expression was confirmed by RT-PCR (3 MOI led to a 18-fold increase in CaMKII expression and 10 MOI to a 114-fold increase).
To further support these data, we asked whether CaMKII is also required for CCL2 and CCL3 gene expression in isolated cardiomyocytes. Thus, we isolated adult mouse ventricular myocytes (AMVMs) from FFFF and DKO and applied hypoxia followed by 8 hours normoxia (Figure 6D). We found that CCL3 but not CCL2 was significantly up-regulated by hypoxia, which was clearly attenuated in DKO cardiomyocytes. Taken together, CaMKII is sufficient and required for cardiomyocyte autonomous CCL3 expression. To test whether an increased gene expression of CCL2 and CCL3 results in an increased secretion of the protein, we performed hypoxia experiments in NRVMs (Figure 6E + F) and found that an increase in CCL2 and CCL3 mRNA preceded an actual increase of CCL2 and CCL3 protein in the supernatant of NRVMs 24 hours after hypoxia, potentially explaining the late effects on infiltration of CD45+ cells."

Response to Referee #3

The quality of Tunel assays is in general very poor throughout the manuscript, raising doubts about the conclusions.

We apologize that the quality of the TUNEL stainings appeared poor in the initial version of the figures. We suspect that this was due to the low resolution of the figures in the word document. In this revised version we provide high resolution images. Actually, the quality of our TUNEL stainings are quite sufficient because the appropriate controls were included: 1) We used sham controls to have a surgery-related negative control in each group. This important control was actually missing in similar investigation by Ling et al. (Ling et al, 2013). 2) We used also the appropriate positive and negative controls for each series of staining. We used pretreatment with DNase I for positive controls and left out TUNEL enzyme solution for negative controls. Again, these controls were missing in the study mentioned before (Ling et al, 2013). Furthermore, we did not exclusively rely on TUNEL stainings but we performed also Caspase activity measurements. With this additional technique we confirmed the negative data derived from TUNEL assays. Moreover, we also included more general markers for cell death and measured high sensitive serum Troponin T levels as an independent surrogate marker of myocardial cell death. Again, no significant changes could be detected by this method.

The western blots in supplemental material need improvement, especially referring to CaMKII delta B KO.

Suppl. Fig 5C shows Western blot analysis of CaMKIIδ splice variant expression, 24 hours after I/R injury in AAV9-CaMKIIδB/C-expressing CaMKIIδ-KO mice. The reason we show this blot in the supplement is to confirm expression of AAV9-mediated splice variant expression. This blot clearly demonstrates expression of CaMKII deltaB splice variant (appr. 56 KDa, left), and deltaC splice variant (appr. 54 KDa, right), respectively. To further strengthen this part, we added new Western Blots (Suppl. Fig. 5D), that show that in the delta B KO only the delta C band appears whereas in the delta C KO only the delta b band appears. To further prove that the splice variant re-expression results in functional CaMKII, we show in the same figure that phospholamban dephosphorylation delta KO is restored by AAV-mediated re-expression of both splice variants.

CaMKIIdelta does not regulate myocardial damage shortly after I/R injury. The conclusion of these results is based upon the TUNEL assays in Fig. 1. According to the authors, the results support that genetic deletion of CaMKII delta does not exert any effect on apoptosis following ischemia-reperfusion damage. It has been extensively and convincingly demonstrated (Brown, 2008; Ling et al, 2013) that genetic deletion of CaMKII delta isoform reduces apoptosis in the same model: the authors do not provide sufficient insights to explain the discrepancies, also showing technical weaknesses into the Tunel assays.

We are aware of the two studies that the reviewer brings to our attention. For this reason, we performed extensive experiments in different genetic CaMKII models that were established for this study (delta KO, splice variant re-expression, double KO, inducible double KO). Off note, we did not use "the same model" as the reviewer states. We used another knockout model that was generated by a different knockout strategy, as discussed already in the first version of the manuscript (see also below). Moreover and as mentioned above, we used different techniques to carefully study immediate cell death mechanisms (not only TUNEL stainings, but also caspase assays and high sensitive troponin T assays) to quantify cell death in hearts from mice that underwent I/R injury. Despite this effort, which is also acknowledged by the other 2 reviewers, we did not find any significant differences between the groups in infarct size or cell death at 24 hours after I/R injury. Neither CaMKII delta single KO, nor specific deletion of CaMKII delta splice variants, nor iDKO, nor DKO did reveal any differences in infarct size. Indeed, it has been demonstrated by Ling et al. that genetic deletion of CaMKII delta isoform reduces apoptosis in the CaMKII delta KO mouse model. Although we used the same protocol of 60 minutes of LAD ligation followed by 24 hours of reperfusion, we could not demonstrate beneficial effects of CaMKII deletion in the acute setting. Thus, we discuss in detail the different approaches that were used to generate the KO mouse models by Ling (Ling et al. 2009) and by us (Backs et al. 2009):

"A possible explanation for these contradictory results could be found in different genetic backgrounds or different KO strategies. Deletion of CaMKIIδ and CaMKIIγ in the present study was achieved by targeting exons 1 and 2 of the two CaMKII genes, resulting in no residual translation of partial CaMKII peptides (Backs et al, 2009). In contrast, the other CaMKIIδ-KO model (Ling et al, 2013; Ling et al, 2009) was generated by targeting exons 9 to 11. Thus, the possibility exists that a residual N-terminal CaMKII polypeptide is produced which exerts a dominant-negative effect on CaMKIIγ or modulatory effects on other kinases because the N-terminal part would be predicted to bind to CaMKII phospho-sites (which are often shared with other kinases) but would not direct kinase activity to these sites."

Role of CaMKIIdelta splice Variants in Acute Myocardial I/R Injury. Fig. 2C and F. The authors need to show controls of void adeno-associated vectors. Furthermore, delta-C CaMKII overexpression has been previously demonstrated to be associated with dilated myocardiopathy (Sossalla, 2011), whereas overexpression of delta-B CaMKII has been demonstrated to associate with increased apoptosis. In both cases, the authors do not address the discrepancy of their results.

We are aware of the aforementioned studies. Besides the association study of Sosalla, the first study showing that CaMKIIdeltaC transgenic mice develop dilated cardiomyopathy was published by Zhang et al. in 2003 (Zhang et al. 2003). In this study it was stated that the two different CaMKII delta C transgenic mouse lines showed 12 and 17-fold overexpression as compared to endogenous CaMKII. One needs to be careful to interpret these findings because of the artificially high expression levels delta C. Likewise, in CaMKII delta B transgenic mice, CaMKII was at least 10-fold overexpressed. Therefore, unphysiological effects could have been obtained due to overexpression artefacts. In contrast to these studies, we expressed CaMKII deltaB and deltaC via AAV9-mediated gene transfer close to the endogenous expression levels in the CaMKII delta KO background – which resulted in a specific deletion of the non-expressed counterpart. To our knowledge, this approach is unique so far and provides the opportunity to investigate the splice variant-specific functions of CaMKII. To reveal expression levels close to endogenous expression levels, we performed titration experiments as shown in Suppl. Fig 5A.The problematic situation with the transgenic approaches is now shortly mentioned in the discussion on page 11:

"Thus, we were interested to test whether the specific lack of CaMKIIδC protects the heart against acute myocardial I/R injury. We took advantage of the AAV9-mediated expression approach to re-express the CaMKII splice variants at endogenous rather than at artificial overexpression levels. In this regard, CMKIIδB and CMKIIδC transgenic mice showed at least a 10-fold overexpression of CaMKII in the heart (Zhang et al, 2002; Zhang et al, 2003), which exceeds the expression levels as observed in pathological conditions (see Figure 2)."

Combined cardiomyocyte-specific deletion of CaMKIIdelta/gamma;does not affect I/R injury. The authors show results (Fig. 3) supporting that PLB phosphorylation is reduced only when they

double knock delta and gamma CaMKII isoforms, whereas no reduction whatsoever is achieved by CaMKII delta, the main isoform expressed in their model. The authors should provide data on isolated cardiomyocite rather than on whole ventricular extract, and should measure CaMKII activity by alternative techniques (for example kinase assays).

We used whole ventricular extracts for PLB phosphorylation because PLB is a cardiomyocyte-specific protein. However, we thank the reviewer for the suggestion and we also isolated adult cardiac myocytes to do the requested experiment. However, we chose to measure in this experiment again PLB phosphorylation, since this is a very specific read out. Over the last years we tested many approaches to detect CaMKII activity. Because we have the advantage to use double CaMKII KO mice as controls, we are able to better judge the specificity of different approaches. Please note, Fig. 3 shows convincingly that only the deletion of both isoforms results in a clear loss of PLB Thr-17 phosphorylation. Thus, this model is so far unique to specifically study a complete loss of CaMKII function. [Unpublished data and figure removed as requested by the Authors].

Fig. 4, Fig. 5 and Fig. 6 all lack the wild type controls: no conclusions can be convincingly drawn by these results.

We used Cre negative littermates of DKO as control (FFFF, stands for four floxed alleles) in Figure 4,5 and 6. As shown in Fig. 3 (see also below), FFFF mice show normal expression of CaMKII and normal phosphorylation levels of PLB (as compared to WT). The use of Cre neg. littermates is the appropriate for these kind of experiments.

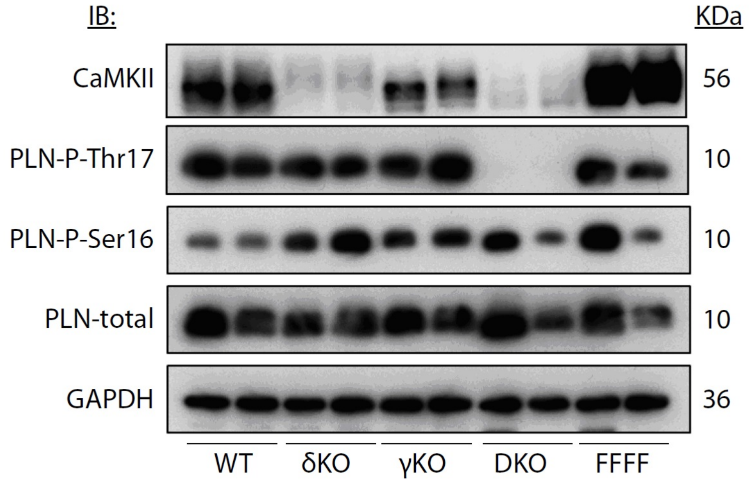

References:

Agah R, Frenkel PA, French BA, Michael LH, Overbeek PA, Schneider MD (1997) Gene recombination in postmitotic cells. Targeted expression of Cre recombinase provokes cardiac-restricted, site-specific rearrangement in adult ventricular muscle in vivo. *J Clin Invest* 100: 169-179

Backs J, Backs T, Neef S, Kreusser MM, Lehmann LH, Patrick DM, Grueter CE, Qi X, Richardson JA, Hill JA, Katus HA, Bassel-Duby R, Maier LS, Olson EN (2009) The delta isoform of CaM kinase II is required for pathological cardiac hypertrophy and remodeling after pressure overload. *Proceedings of the National Academy of Sciences of the United States of America* 106: 2342-2347

Ling H, Gray CB, Zambon AC, Grimm M, Gu Y, Dalton N, Purcell NH, Peterson K, Brown JH (2013) Ca2+/Calmodulin-dependent protein kinase II delta mediates myocardial ischemia/reperfusion injury through nuclear factor-kappaB. *Circ Res* 112: 935-944

Ling H, Zhang T, Pereira L, Means CK, Cheng H, Gu Y, Dalton ND, Peterson KL, Chen J, Bers D, Heller Brown J (2009) Requirement for Ca2+/calmodulin-dependent kinase II in the transition from pressure overload-induced cardiac hypertrophy to heart failure in mice. *J Clin Invest* 119: 1230-1240

Subramanian A, Tamayo P, Mootha VK, Mukherjee S, Ebert BL, Gillette MA, Paulovich A, Pomeroy SL, Golub TR, Lander ES, Mesirov JP (2005) Gene set enrichment analysis: a knowledge-based approach for interpreting genome-wide expression profiles. *Proc Natl Acad Sci U S A* 102: 15545-15550

Zhang T, Johnson EN, Gu Y, Morissette MR, Sah VP, Gigena MS, Belke DD, Dillmann WH, Rogers TB, Schulman H, Ross J, Jr., Brown JH (2002) The cardiac-specific nuclear delta(B) isoform of Ca2+/calmodulin-dependent protein kinase II induces hypertrophy and dilated cardiomyopathy associated with increased protein phosphatase 2A activity. *J Biol Chem* 277: 1261-1267

Zhang T, Maier LS, Dalton ND, Miyamoto S, Ross J, Jr., Bers DM, Brown JH (2003) The deltaC isoform of CaMKII is activated in cardiac hypertrophy and induces dilated cardiomyopathy and heart failure. *Circ Res* 92: 912-919

2nd Editorial Decision 13 July 2014

Thank you for the submission of your revised manuscript to EMBO Molecular Medicine. We have now received the enclosed reports from the referees that were asked to re-assess it. As you will see the reviewers are now globally supportive and I am pleased to inform you that we will be able to accept your manuscript pending the following final amendments:

1) Please provide an accession number for your submitted gene array data.

2) Every published paper now includes a 'Synopsis' to further enhance discoverability. Synopses are displayed on the journal webpage and are freely accessible to all readers. They include a short standfirst - to be written by the editor - as well as 2-5 one sentence bullet points that summarise the paper (to be written by the author). Please provide the short list of bullet points that summarise the key NEW findings. The bullet points should be designed to be complementary to the abstract - i.e. not repeat the same text. We encourage inclusion of key acronyms and quantitative information. Please use the passive voice. Please attach these in a separate file or send them by email, we will incorporate them accordingly.

3) We are now encouraging the publication of source data, particularly for electrophoretic gels and blots, with the aim of making primary data more accessible and transparent to the reader. Would you be willing to provide a PDF file per figure that contains the original, uncropped and unprocessed scans of all or at least the key gels used in the manuscript? The PDF files should be labeled with the appropriate figure/panel number, and should have molecular weight markers; further annotation may be useful but is not essential. The PDF files will be published online with the article as supplementary "Source Data" files. If you have any questions regarding this just contact me.

Please submit your revised manuscript within two weeks.

I look forward to reading a new revised version of your manuscript as soon as possible.

***** Reviewer's comments *****

Referee #1 (Comments on Novelty/Model System):

This revised manuscript improved significantly from the initial submission.

Referee #1 (Remarks):

The authors address my comment adequately. I have no further comments/suggestions.

Referee #2 (Remarks):

The author responded adequately to my comments. no further questions

Referee #3 (Comments on Novelty/Model System):

I really appreciated the efforts made by the authors, that resulted in an improved manuscript. They not only enhanced the overall quality of the figures, but also overcame some weak points in the manuscript by adding new experiments.

This version is now acceptable for publication

2nd Revision - authors' response 28 July 2014

We would like to thank you for your willingness to accept our manuscript. Today we submit the final amendments that you requested:

1. The accession number for our submitted gene array: GEO-Nummer: GSE58486
2. Short synopsis (4 sentences):

   a) Acute myocardial I/R-induced damage is not mediated by CaMKII

   b) Post-I/R remodelling and inflammatory processes are mediated by CaMKII. In particular,

   c) Leukocyte infiltration and expression of members of the chemokine (C-C motif) ligand family, in particular CCL3 (macrophage inflammatory protein-1α, MIP-1α), are reduced in mice lacking the two cardiac CaMKII isoforms delta and gamma upon I/R injury.

   d) CaMKII is sufficient and required for CCL3 expression in cardiomyocytes
3. Original, uncropped and unprocessed scans of the key gels used in the manuscript: We now uploaded a pdf file with "uncut gels" for Fig. 2 and 3, as well as Supplemental Figures 4,5A, 5C, 5D, 6 and 7B

We also made some minor modifications to the acknowledgement (SFB 1118) and some changes to the format in the supplement (Suppl. Fig. 9) for a clearer view on the data. We did not change any content of the manuscript as compared to the last submission.

We request to upload a slightly modified version of the rebuttal letter for the online version because we included some unpublished data (Fig. R1 and upper three panels of Fig. R2) from two other manuscripts to clarify two points in the communication with the reviewers. We did not change other parts of the rebuttal letter. So, we request to use the modified version for the online publication of our rebuttal.

3rd Editorial Decision 29 July 2014

Everything appears Ok now but for one thing. This is in part my fault as I failed to mention it in my recent decision letter: As per our Author Guidelines, the description of all reported data that includes statistical testing must state the name of the statistical test used to generate error bars and P values, the number (n) of independent experiments underlying each data point (not replicate measures of one sample), and the actual P value for each test (not merely 'significant' or 'P < 0.05'). You may insert the proper values directly in the figure legends (manuscript and supplemental file). Please upload the manuscript files without any highlighting as I have now seen the modifications.

Again, sorry for not spotting this omission earlier and please submit your revised manuscript as soon as possible. so that I may proceed with formal acceptance.

3rd Revision - authors' response 30 July 2014

Enclosed is a revised manuscript entitled “CaM Kinase II mediates maladaptive post-infarct remodeling and pro-inflammatory chemoattractant signaling but not acute myocardial ischemia/reperfusion injury” by Weinreuter et al. that we are re-submitting for consideration for publication in *EMBO Mol Med*.

We would like to thank you for your willingness to accept our manuscript. Today we submit the final amendments that you requested. We also made some minor modifications to the acknowledgement and some changes to the format in the supplement (Suppl. Fig. 9) for a clearer view on the data. We did not change any content of the manuscript as compared to the last submission.
